# The potential for biochar application in “Shatangju” (*Citrus reticulate* cv.) orchard on acid red soil: Biochar prepared from its organic waste in an orchard

**DOI:** 10.3389/fpls.2022.1001740

**Published:** 2022-10-20

**Authors:** Yanjun Guo, Baoli Qiu, Zaid Khan, Hui Jiang, Qianhua Ji, Qizhou Fan, Muhammad Musa Khan

**Affiliations:** ^1^ Fruit Tree Research Institute/Life Sciences College of Zhaoqing University, Zhaoqing, China; ^2^ Chongqing Key Laboratory of Vector Insects, College of Life Sciences, Chongqing Normal University, Chongqing, China; ^3^ College of Natural Resources and Environment, South China Agricultural University, Guangzhou, China; ^4^ Engineering College of Huazhong Agricultural University, Wuhan, China; ^5^ Hainan Institute of Zhejiang University, Sanya, China

**Keywords:** biochar, *Citrus reticulate*, acid red soil, organic carbon cycle, soil microbiota, nitrogen content, fruit quality

## Abstract

Carbonization of agricultural and forestry wastes is the main use of biochar application in agriculture. In this study, the effects of biochar on the physical and chemical properties of soil and diversity in rhizosphere microorganisms, leaf nutrients and fruit quality of acid red soil in “Shatangju” (*Citrus reticulate* cv.) orchard were studied using organic wastes and small-scale carbonization furnaces from orchards were used to produce biochar. The results showed that the finished rate of biochar produced from the organic wastes in the orchard was approximately 37%, and the carbon content of the finished product was as high as 80%. The results suggested that the biochar produced in the orchard could meet the annual consumption of the orchard. Applying biochar can improve the physical and chemical properties of acid soil in the “Shatangju” orchard by enhancing the availability of various mineral nutrients such as nitrogen, phosphorus, potassium, calcium, magnesium and boron. The species and quantity of root and rhizosphere microbial communities (fungi, bacteria and archaea) increased, and the dominant bacterial group changed, manifested in the increase in microbial diversity. Biochar directly affected the soil pH value and increased the soil organic carbon content, which may be the main reason for the change in microbial diversity in the soil and rhizosphere of “Shatangju” in the orchard and pot tests. The fruit quality of each treatment group with biochar was also better than that of the control group and improved fruit coloring. In the pure soil test, whether or not chemical fertilizer was applied, 3% biochar amendments can provide a suitable pH value for “Shatangju” growth and are relatively stable. Regardless of whether or not fertilizer was applied, 1.5%-3% biochar improved the soil in the pot test. In the field, the biochar at a rate of 2.4 kg/plant to 3.6 kg/plant, respectively, was the best in improving soil physical and chemical properties, foliar nutrition and fruit quality. Therefore, the amount of biochar added in the open environment (if the garden) can be slightly adjusted according to the results of the closed environment test (pure soil test and pot test). In this experiment, we explored the self-recycling of organic carbon, mainly through the preparation of a simple small-scale biochar furnace suitable for the use by orchards, and selected the appropriate amount of biochar to improve the physical and chemical conditions of “Shatangju” orchard soil and increase fruit quality.

## 1 Introduction

Agricultural practices improve soil quality and increase soil nutrient supply capacity, which has been emphasized in many fields across basic and applied sciences (Puget and Lal, 2005; Du et al., 2013; Iqbal et al., 2022). Fertilizers are important agricultural inputs that play an irreplaceable role in agricultural development. Excessive chemical fertilizer application is a common and serious problem in China as farmers are concerned about increased food demand, low soil fertility, and a high multiple cropping index (Tian et al., 2016). Excessive chemical fertilizer application reduces soil quality, acidification, and nonpoint source pollution (Xia et al., 2020). Thus, controlling chemical fertilizer application while ensuring high and stable yield is a key area of agricultural research. Limited use of organic nutrient resources and application of organic materials to replace some chemical fertilizers have been shown to effectively reduce the amount of chemical fertilizer needed, increase soil productivity, ensure crop yield, and improve local ecological functions (Liu et al., 2022).

Carbonization of agricultural and forestry wastes is the focus of biochar application in agriculture (Pawlak-Kruczek et al., 2020). Biochar is a carbon (C) rich thermally decomposed organic material produced through the pyrolysis process from the feedstock at high temperatures (300°C-700°C) under a limited supply of oxygen (Zhang et al., 2013b). The feedstock may include crop residues, tree bark, wood materials, chicken litter, sewage sludge or dairy manure (Oshunsanya and Aliku, 2016). It is important to mention that biochar differs from charcoal and carbon-based materials. It has distinctive biological, chemical and physical properties (Oni et al., 2019). Its porous structure, high surface area, and low degradation rate played an important role in soil nutrient retention (Khan et al., 2021b). The high pH, electric conductivity (EC), cation exchange capacity (CEC), high carbon contents and abundance surface functional groups are important for environmental pollutant complexation, e.g., heavy metals (Ahmad et al., 2014; Bashir et al., 2018). Incorporating biochar in the soil can prominently increase soil aeration, porosity, water holding capacity and nutrients, which improve soil fertility, plant growth and carbon sequestration in soil. The high pH of biochar is due to its high alkalinity and CEC, which can enhance soil efficiency (Ahmad et al., 2014).

Soil microorganisms play an imperative role in the nutrient cycle of soil, including the organic matter composition and soil aggregate arrangement (Oleszczuk et al., 2014). Soil microorganisms affect soil fertility and ecosystems (Tardy et al., 2015). Numerous studies have recorded an enhancement in soil microorganisms and biomass by adding biochar to soil (Bruun et al., 2014; Yu et al., 2019). Meng et al. (2019) documented that biochar derived from wheat straw incorporation into soil significantly increased the community abundance and diversity of plant beneficial bacterial and fungal taxa and the variety of plant wheat seedling rhizospheres. Additionally, Ahmad et al. (2016) described that the microbiota in the applied soil was significantly diverse, e.g., Pseudomonas, a major rhizosphere-encouraging bacteria, was markedly improved by the biochar applied treatments. Biochar may likewise enhance the abundance of ammonia-oxidizing bacteria and archaea and reduce (Proteobacteria, Firmicutes) the overall abundance of oligotrophic and copiotrophic taxa separately (Meng et al., 2019). On the other hand, biochar application was also reported to reduce abundance of microbial communities of Proteobacteria, Acidobacteria, Firmicutes, and Bacteroidetes (Wu et al., 2016).

“Shatangju” is China’s main citrus variety and a famous distinct local variety (Wang et al., 2012b). It is characterized by easy peeling, no core, slagging and high sweetness (Li et al., 2012; Li et al., 2019). Recently, it has been mainly grown in Guangdong Province and Guangxi Province, belonging to the citrus industrial belt in the Xijiang River Basin of China. The “Shatangju” orchard covers an area of 6 million mu (Chaisiri et al., 2021). The soil in the main planting area of “Shatangju” is the acidic red soil. Fruit farmers generally use lime to improve the soil acidity with lime. However, a large amount of long-term application of lime will cause soil hardening, damage beneficial soil microorganisms, reduce soil organic carbon, and easily cause the imbalance of soil calcium, potassium, magnesium and other elements, and the soil is prone to acid reversion (Zhang et al., 2013c).

In China, relevant research on biochar was conducted, and there are many fields of application, but there are few examples related to production of “Shatangju.” This study was designed to evaluate the effect of biochar prepared from “Shatangju” orchard waste on improving the properties of acid red soil and affecting the yield and quality of “Shatangju.” The effect of “Shatangju” plant waste biochar on the soil physical and chemical properties of soil was explored in this study. The composition of soil and root microbial communities under different biochar amendments was also examined. At the same time, the effect of biochar on fruit quality was evaluated. The results of this study will provide information about self-made biochar under field conditions for improving “Shatangju” production.

## 2 Materials and methods

### 2.1 Plant material

The experiments were conducted in 2020 and 2021. The “Shatangju” plants (2 years old) plants were grown in root-pruning bags with a diameter of 60 cm and height of 40 cm filled with acid red soil and were used for pot experimentation; ten-year-old “Shatangju” plants were used as the experimental trees in the orchard, with row spacing of 2.5 m × 2.5 m. The acid red soil bearing the following physiochemical was used as potting soil: pH 4.43, OMC (Organic matter content) 15.17 g/kg, A-N (available nitrogen) 72.57 mg/kg, A-P (available phosphorus) 15.23 mg/kg, A-K (available potassium) 369.93 mg/kg, E-Ca (exchangeable calcium)1.82 g/kg, E-Mg (exchangeable magnesium) 0.15 g/kg, A-Zn (available zinc) 18.79 mg/kg, A-B (available boron)1.17 mg/kg, A-Cu (available copper) 0.54 mg/kg and E-Mn (exchangeable manganese) 18.57 g/kg. Orchard soil: moisture content 19.9%, field capacity 30%, bulk density1.22 g/cm, porosity 3.65%, pH4.82, OMC 21.48 g/kg, CEC 8.04 cmol/kg, A-N 110.52 mg/kg, A-P 22.71 mg/kg, A-K 153 mg/kg, E-Ca 889.45mg/kg, E-Mg 49.9 g/kg, A-Zn 3.88 mg/kg, and A-B0.86 mg/kg.

### 2.2 Test method

#### 2.2.1 Preparation and index determination of biochar

Biochar was prepared from pruned citrus branches and interrow grass in the citrus orchard using a Shizishan brand carbonization furnace provided by the School of Engineering, Huazhong Agricultural University, Patent # ZL 201310290114.X. The branches were cut into 10-cm and 20-cm pieces after pyrolysis at 500°C-550°C under anoxic conditions. Biochar was screened through a 20-mesh sieve and used in the pot and field experiments. Plant ash was prepared with the same materials as the control. The samples’ yield rate, moisture content, ash content, and carbon content of the samples were measured (Brahmakshatriya and Donker, 1971; Wang et al., 2009). The experiment was repeated three times every year.

The effect of biochar on the pH value of red soil was determined by pouring 2 kg of acid red soil into each flower pot. Biochar was applied to the flowerpot according to 0%, 1%, 2%, 3%, 4%, 5% and 6% of the soil weight. NPK 1% (15-15-15) compound fertilizer was prepared and applied in a flowerpot containing biochar. The soil pH value was measured continuously throughout the soil incubation period. The experiment was repeated three times every year.

#### 2.2.2 Biochar amendments on citrus in pot tests

The 25 kg crushed and air-dried red soil was spiked with biochar and placed in 35 L root-pruning bags. One “Shatangju” was planted in a bag in rain-shelter cultivation conditions. The percentages of biochar amendments to the dry weight of red soil were 0, 1.5%, 3%, 4.5%, and 6%. The base fertilizer was modified Hoagland-Amon nutrient solution [KH_2_PO_4_, KNO_3_, Ca (NO_3_)_2,_ and MgSO_4_.7H_2_O were 136, 505, 1180, and 492 mg/kg, respectively; H_3_BO_3_, MnCl.4H_2_O, ZnSO_4_.7H_2_O, CuSO_4_.5H_2_O, Na_2_MoO_4_ and EDTA-Fe were 2.86, 1.81, 0.22, 0.08, 0.09 and 48.5 mg/kg, respectively]. The experiment was repeated three times every year.

Including the control, ten treatments were established: 1.5% biochar (1.5% C), 3% biochar (3% C), 4.5% biochar (4.5% C), 6% biochar (6% C), base fertilizer (BF), base fertilizer + 1.5% biochar (BF+1.5% C), base fertilizer + 3% biochar (BF+3% C), base fertilizer + 4.5% biochar (BF+4.5% C) and base fertilizer + 6% biochar (BF+6% C). The soil and plant indices were measured at the time of planting and 9 months post-cultivation. In the control group, only the dressing furrow was dug, and the soil was backfilled. The experiment was repeated three times every year.

#### 2.2.3 Biochar amendments on “Shatangju” in the orchard

Six treatments, including 0 kg biochar/plant (control), 1.2 kg biochar/plant (C-1.2 kg), 2.4 kg biochar/plant (C-2.4 kg), 3.6 kg biochar/plant (C-3.6 kg), 4.8 kg biochar/plant (C-4.8 kg) and 6 kg biochar/plant (C-6 kg), were established. The location of biochar application was at the drip line of the tree crown. One 80 cm long, 30 cm wide, and 30 cm deep dressing furrow was dug from the north and the south and the biochar and soil were mixed evenly before the application.

### 2.3 Sample collection and determination method

#### 2.3.1 Soil and root samples

In the pot experiments, 0-20 cm topsoil at 5-10 cm away from the plant trunk was collected. In the orchard experiment, two biochar amendment sites were randomly selected, and the soil 0-30 cm deep from the soil layer was evenly collected. The quartering method was used diagonally, choosing 0.5 kg soil samples to determine the soil pH value and available nutrients. The roots and rhizosphere soil were frozen at -80°C, and the microbial community diversity of the soil, rhizosphere soil, and roots was measured.

The drying method was used for soil moisture content measurement, the Wilcox method for field capacity, and the ring-cutting method for bulk density (Khan et al., 2022). The pH value was determined by potentiometry (soil water ratio was 1:2.5), and soil organic matter was analyzed by oxidation by a saturated potassium dichromate solution with oil bath heating. The EDTA ammonium acetate exchange was used to determine the CEC. Available nitrogen was determined by the alkaline hydrolysis diffusion method, available phosphorus was determined by ammonium fluoride-hydrochloric acid by using extraction molybdenum antimony resistance colorimetric method, available potassium was determined by neutral ammonium acetate extraction-flame photometry, exchangeable calcium and magnesium were extracted by ammonium acetate and determined usnig atomic absorption spectrometry, available zinc was determined by DTPA extraction-atomic absorption spectrometry, and available boron was determined by boiling water extraction curcumin colorimetric (Mikula et al., 2020). The standard soil sample was GBW07417a (ASA-6a) from paddy soil in Guangdong Province.

The detection of microbial community diversity in the roots, rhizosphere, and soil was as follows. Genomic DNA was extracted for PCR amplification, and specific primers with barcodes were designed according to the designated sequencing region. Library construction and Illumina PE250 sequencing, OTU cluster analysis, and species taxonomy analysis were conducted to determine microbial community diversity, relative abundance and the community structure component diagram.

#### 2.3.2 Leaf samples

From the tree canopy, the second to fourth intact and healthy leaves were collected from the top of underyearling vegetative spring shoots from all four directions. Three leaves were collected from each direction, which was repeated twice for each direction. Leaves were mixed in a plastic bag, and 24 leaves were chosen and brought back to the laboratory to determine nutrient content.

Total nitrogen was determined by the H_2_SO_4_-H_2_O_2_ Kjeldahl digestion method. Total potassium was determined by H_2_SO_4_-H_2_O_2_ Kjeldahl digestion and the molybdenum antimony resistance colorimetric method, total potassium was determined by H_2_SO_4_-H_2_O_2_ Kjeldahl digestion and flame photometry (Khan et al., 2022), and total calcium, magnesium, and zinc were determined by dry ashing-dilute hydrochloric acid dissolution atomic absorption spectrometry. Total boron was determined by dry ashing-dilute hydrochloric acid dissolution curcumin colorimetry (Bao, 2005).

#### 2.3.3 Fruit samples

At the stage of maturation and harvesting, twelve fruits were randomly selected from each tree from all four directions around the upper part of the crown, and the single fruit weight and quality were determined.

The fruit uniformity was determined by the proportion of medium fruit (diameter of 36.5-42 mm) (Wang et al., 2012b). The color difference de, brightness L, redness a, and yellowness b of the peel were determined by a Minolta CR-300 automatic colorimeter. The fruit shape index was the ratio of longitudinal diameter to transverse diameter. The peel thickness was measured with a Vernier caliper according to the cross-section of the equatorial line. The solid soluble content was measured using the RA-250 WE digital sugar meter (KEM company, Japan). The titratable acid content was measured using the NaOH neutralization titration method (GB/T 12456-2008). Reducing sugar and total sugar were determined by 3,5-dinitrosalicylic acid colorimetry (Li, 2000). Vitamin C content was determined by the 2,6-dichlorophenol indophenol (DCPIP) titration method (GB/T 5009.86-2016).

### 2.4 Data analysis

The data were sorted out in Excel 2007. The differences between each treatment and the control were analyzed by t test; Duncan’s multiple comparison method was used to analyze the significance of the differences among the treatments at *P*<0.05. The analysis was conducted with six replications (3 replications per year) The graphs of different experiments were made using Graphpad Prism 5. The test data were analyzed by SPSS (25) for Windows software.

## 3 Results

### 3.1 Optimization of biochar preparation conditions and comparative analysis

**Figure 1:**
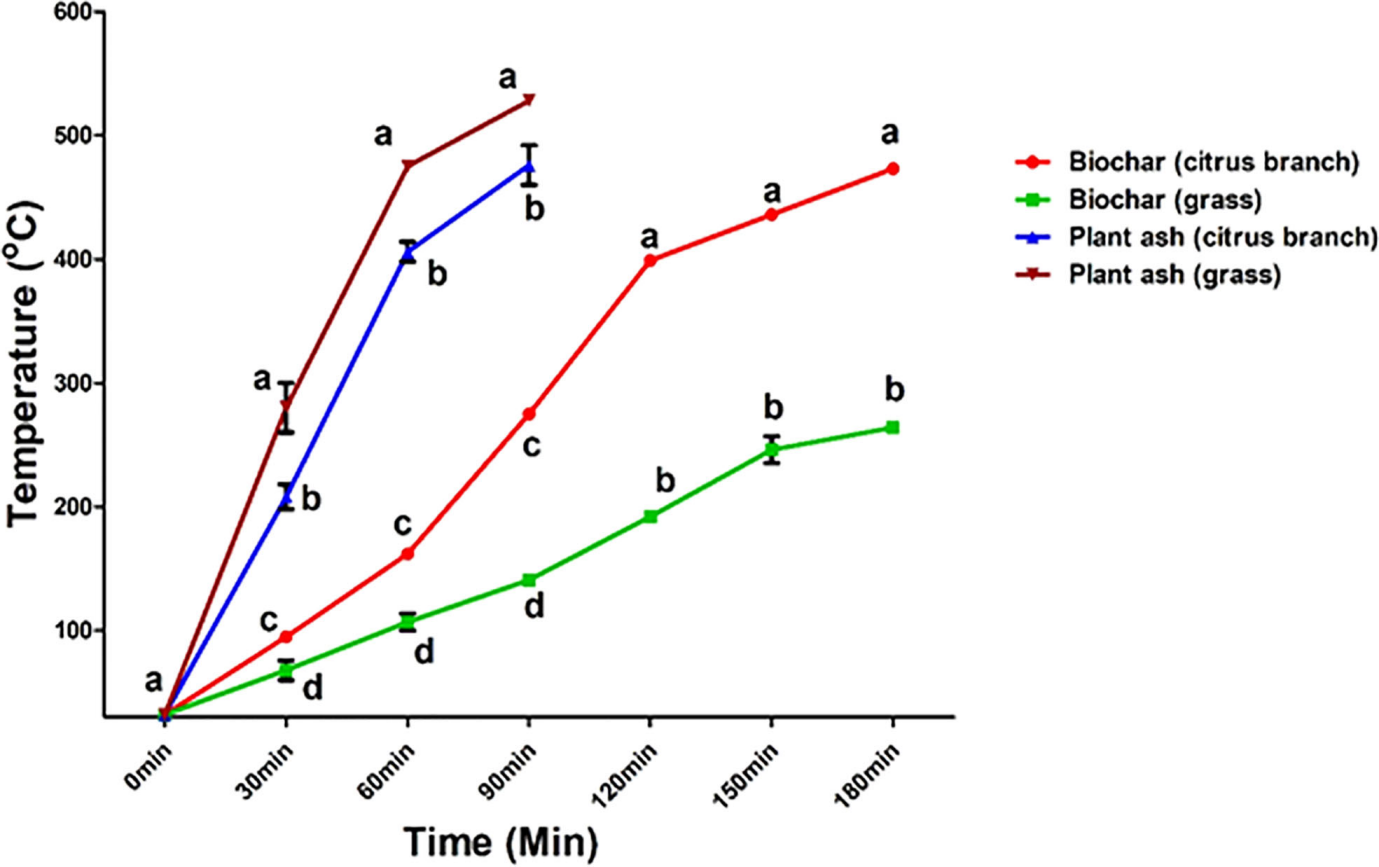
Response of time and temperature to various types of citrus branch and grass during biochar preparation. Lowercase letters show the difference among different temperatures at a specific time at *P*<0.05. Lowercase letters on a specific time have no significant difference. Error bars represent the standard deviation of the mean.

During the process of biochar preparation (**Figure 1**), the heating rate was affected by the type and length of the materials. The heating rate of grass was much higher than that of the “Shatangju” branches. The shorter the length the “Shatangju” branch was, the faster the heating rate was. After heating for 1 hour, the two began to show significant differences. The biochar yield of grass was slightly higher than that of the “Shatangju” branches, and the biochar yield of the10-cm raw material was higher than that of the 20-cm raw material, but the difference did not reach a significant level. The carbon content in biochar from Citrus branches was significantly higher than that in biochar from grass (**Table 1**). Different proportions of biochar amendments could increase the pH value of acid red soil, and with increasing time, the pH value increased and remained relatively stable later (**Table 2**). When chemical fertilizer was applied to the red soil with biochar amendments (**Table 3**), the pH value of the soil in the sample groups with 3%, 4%, 5% and 6% biochar showed a decreasing trend. The pH value of red soil decreased slightly, but the change was not obvious in the sample groups with 1% and 2% biochar. With continuous fertilizer application, the pH value of the soil was maintained at approximately 5.5 in the sample group with 3% biochar, and the fluctuation range of pH value was smaller than that of the sample group with 4%, 5% and 6% biochar. Therefore, the pH of acid red soil with 3% biochar was improved and remained stable for a long time. This pH value (5.5) was close to that suitable for citrus growth.

**Table 1:** Characteristics of biochar prepared by different materials.

Raw material	Yield (%)	Moisture (%)	Ash (%)	Carbon (%)
Biochar (citrus branch)	37.00±2.45 a	5.30±0.10 a	13.0±2.0 c	81.7±0.2 a
Biochar (grass)	39.00±1.50 a	5.07±0.07 a	20.3±0.3 c	74.7±0.7 b
Plant ash (citrus branch)	33.10±0.10 b	4.50±0.15 b	85.0±3.0 a	09.7±0.6 d
Plant ash (grass)	41.70±1.70 a	1.40±0.00 c	75.0±1.0 b	15.0±0.5 c

The lowercase letters behind the values of the same indicator in each column are different, indicating a significant difference between them at P<0.05. Similar letters indicate no significant difference.

**Table 2:** Effect of different incubation periods on the pH of different concentrations of biochar.

Biochar concentration							
Days of incubation	Control(Mean±SE)	1%(Mean±SE)	2%(Mean±SE)	3%(Mean±SE)	4%(Mean±SE)	5%(Mean±SE)	6%(Mean±SE)
0d	4.65±0.00eA	5.13±0.19dA	5.44±0.04cE	5.87±0.02bE	5.86±0.04bE	6.26±0.01aC	6.30±0.00aD
3d	4.51±0.01fC	5.15±0.01eA	5.48±0.04dDE	5.78±0.00cG	6.09±0.09bD	6.23±0.01bC	6.45±0.05aC
10d	4.60±0.00gA	5.2±0.00fA	5.56±0.00eD	6.27±0.01dC	6.09±0.01cD	6.79±0.09bB	7.15±0.00aA
17d	4.52±0.03gBC	5.21±0.01fA	5.65±0.00eC	6.22±0.02dD	6.33±0.03cBC	6.70±0.00bB	6.94±0.05aB
24d	4.58±0.00eAB	5.37±0.03dA	5.91±0.01cA	6.68±0.01bA	6.69±0.01bA	7.16±0.01aA	7.16±0.01aA
31d	4.53±0.01eBC	5.33±0.03dA	5.92±0.02cA	6.33±0.02bB	6.29±0.06bC	6.99±0.10aA	7.11±0.00aA
38d	4.50±0.03gC	5.21±0.02fA	5.82±0.02eB	6.15±0.00dE	6.47±0.02cB	6.76±0.06bB	7.16±0.01aA

The lowercase letters behind the values indicating statistical significance of the values in a row, while uppercase values indicating statistical significance of the values in a column at P<0.05. Similar letters indicate no significant difference.

**Table 3:** Effect of fertilization on the pH value of acid red soil with biochar amendments.

Amendment	Control	1%	2%	3%	4%	5%	6%
1d	3.94±0.01b	4.67±0.07b	5.14±0.00b	5.77±0.10a	6.27±0.02a	6.55±0.00a	6.87±0.02a
5d	4.22±0.02a	4.97±0.10a	5.39±0.09a	5.89±0.01a	5.96±0.02b	6.36±0.00b	6.58±0.01b
9d	4.24±0.04a	4.73±0.05ab	5.27±0.02a	5.75±0.07ab	5.87±0.02b	6.15±0.10c	6.32±0.00c
13d	4.25±0.05a	4.83±0.08a	5.14±0.06b	5.55±0.10bc	5.61±0.03c	5.89±0.01d	6.05±0.05d
17d	4.30±0.10a	4.81±0.09a	5.10±0.10b	5.39±0.10c	5.63±0.07c	5.79±0.05e	5.87±0.03e

The lowercase letters behind the values of the same indicator in each column are different, indicating a significant difference between them at P<0.05. Similar letters indicate no significant difference.

### 3.2 Effects of different biochar amendments on potted “Shatangju”

#### 3.2.1 Effects of different biochar amendments on the nutritional contents of leaves of potted “Shatangju”

Before planting, the nutrient contents of leaves were determined as follows: total nitrogen 20.42 g/kg, total phosphorus 2.46 g/kg, total potassium 14.39 g/kg, calcium 9.76 g/kg, magnesium 2.55 g/kg, zinc 26.8 mg/kg, and manganese 63.3 mg/kg. After nine months of cultivation (**Table 4**), the contents of calcium and zinc were significantly increased, the contents of phosphorus, potassium and manganese were decreased, and the contents of nitrogen and magnesium were maintained at the original level. The contents of phosphorus, potassium and calcium increased with biochar amendments.

**Table 4:** Changes in mineral nutrients in leaves of “Shatangju” after nine months of cultivation with different biochar amendments in pot tests.

Amendment	N (g/kg)	P (g/kg)	K (g/kg)	Ca (g/kg)	Mg (g/kg)	Zn (mg/kg)	Mn (mg/kg)
CK	22.82±0.07f	1.12±0.03e	09.18±0.01e	22.96±0.06g	2.48±0.08c	39.49±0.67c	35.65±0.67c
1.5%C	21.33±0.05h	1.62±0.01c	11.14±0.01c	25.55±0.04d	2.09±0.06d	39.00±0.80d	13.22±0.58e
3%C	22.07±0.08g	2.18±0.04a	10.47±0.02d	27.58±0.04c	2.88±0.07a	44.07±0.78b	11.33±0.30fg
4.5%C	21.08±0.04i	2.00±0.12b	11.56±0.02a	23.91±0.02f	2.06±0.03d	37.23±0.52d	10.47±0.38g
6%C	23.07±0.05e	2.01±0.04b	11.48±0.05a	27.98±0.03b	2.14±0.01d	37.14±0.04d	12.36±0.22ef
BF	27.81±0.08a	1.05±0.12e	08.63±0.03f	20.74±0.02h	2.84±0.05a	43.05±0.43b	59.50±0.40a
BF+1.5%C	24.05±0.10d	1.30±0.01d	09.14±0.01e	27.53±0.01c	2.5b±0.07c	40.56±0.19c	51.23±0.24b
BF+3%C	20.22±0.03j	1.60±0.08c	08.11±0.08g	25.44±0.08e	2.46±0.05c	37.72±0.27d	14.95±0.95d
BF+4.5%C	24.85±0.04c	1.55±0.03c	10.49±0.07d	28.12±0.07a	2.15±0.00d	40.76±0.22c	10.55±0.20g
BF+6%C	25.98±0.13b	1.56±0.05c	11.32±0.02b	24.92±0.02f	2.66±0.02b	75.08±0.46a	13.06±0.70e

The lowercase letters behind the values of the same indicator in each column are different, indicating a significant difference between them at P<0.05. Similar letters indicate no significant difference.

#### 3.2.2 Effects of different biochar amendments on the soil of potted “Shatangju”

As shown in **Table 3**, the pH value of the soil increased after biochar amendments, and after adding base fertilizer, the increased range of pH values decreased. With the increase of 1.5% biochar, the pH value increased by approximately 0.26-0.47 units, and the OMC increased by 14-15 g/kg. Except for available manganese, the contents of all mineral elements showed an increasing trend. Among the treatments with biochar only, the change in alkali-hydrolyzed nitrogen was small, As shown in **Table 5**, after nine months of cultivation, the fluctuation range of the soil pH value was not large, and the pH value of each treatment with base fertilizer increased slightly, indicating that the stability of acidic soil improved by biochar was better. The biochar amendments promoted the utilization of organic matter and available boron, and the utilization rates of alkali-hydrolyzable nitrogen, exchangeable calcium and available boron were all high in the treatments with base fertilizer.

**Table 5:** Physicochemical properties of soil in different treatments when planted in pot tests.

Amendment	pH	OMC	A-N	A-P	A-K	E-Ca	E-Mg	A-Zn	A-B	A-Cu	E-Mn
CK	4.43±0.02e	15.17±0.12j	72.57±1.07g	15.23±0.73e	369.93±49.54bc	1.82±0.02g	0.15±0.01f	1.88±0.09f	1.17±0.10cd	0.54±0.04f	18.57±0.07e
1.5%C	5.57±0.02c	46.47±0.47f	74.43±3.63fg	36.84±0.66c	373.17±27.97bc	4.41±0.06e	0.38±0.03e	2.69±0.09c	1.28±0.03bc	0.99±0.05bcd	18.49±0.09e
3%C	5.57±0.03c	50.58±0.62e	76.53±2.73fg	35.37±0.37c	428.76±13.36ab	4.98±0.14d	0.45±0.03de	2.80±0.09c	1.41±0.01ab	0.89±0.02d	18.22±0.22e
4.5%C	5.94±0.03b	75.56±0.76b	78.63±2.73fg	53.06±0.84b	374.79±29.62bc	6.21±0.16c	0.46±0.06de	3.97±0.11b	1.07±0.02de	1.06±0.01ab	17.38±0.39f
6%C	6.35±0.03a	88.07±0.27a	81.43±1.00f	67.79±7.44a	465.46±23.86a	7.94±0.29b	0.56±0.06cd	4.74±0.01a	1.20±0.016bc	1.11±0.01a	22.57±0.07a
BF	3.92±0.07f	17.00±0.10i	156.57±3.58e	25.05±0.48d	267.39±31.49e	3.08±0.07f	0.75±0.03b	2.68±±0.06c	0.98±0.07e	0.91±0.04d	20.13±0.11cd
BF+1.5%C	4.45±0.05e	31.93±0.70h	166.13±0.66d	30.46±0.99	299.23±8.92de	4.45±0.06e	0.67±0.09bc	2.11±0.01d	1.08±0.04de	1.03±0.02abc	18.5±0.09e
BF+3%C	4.91±0.03d	42.47±0.40g	173.83±1.41c	30.95±0.85	348.34±8.02cd	4.85±0.27e	0.62±0.05bc	2.39±0.04d	1.09±0.05de	0.90±0.05d	20.4±0.04c
BF+4.5%C	5.71±0.06c	58.40±0.27d	191.8±1.55b	56.99±1.00b	420.12±8.62bc	5.92±0.18c	0.64±0.02b	1.41±0.05g	1.17±0.06cd	0.93±0.03cd	19.78±0.03d
BF+6%C	5.88±0.10b	73.00±1.00c	206.03±2.51a	55.51±3.00b	416.88±6.08bc	8.02±0.02a	0.96±0.06a	2.24±0.04d	1.44±0.02a	0.62±0.02e	21.84±0.04b

BF: base fertilizer; OMC: Organic matter content; A: Available; E: Exchangeable; for OMC, exchangeable Ca, Mg and Mn, g/kg; for available N, P, K, Zn, B and Cu, mg/kg. The lower case letters behind the values of the same indicator in each column are different, indicating that there is a significant difference between them(P<0.05).

#### 3.2.3 Effects of different biochar amendments on soil and root microbial community diversity of potted “Shatangju”

**Figure 2:**
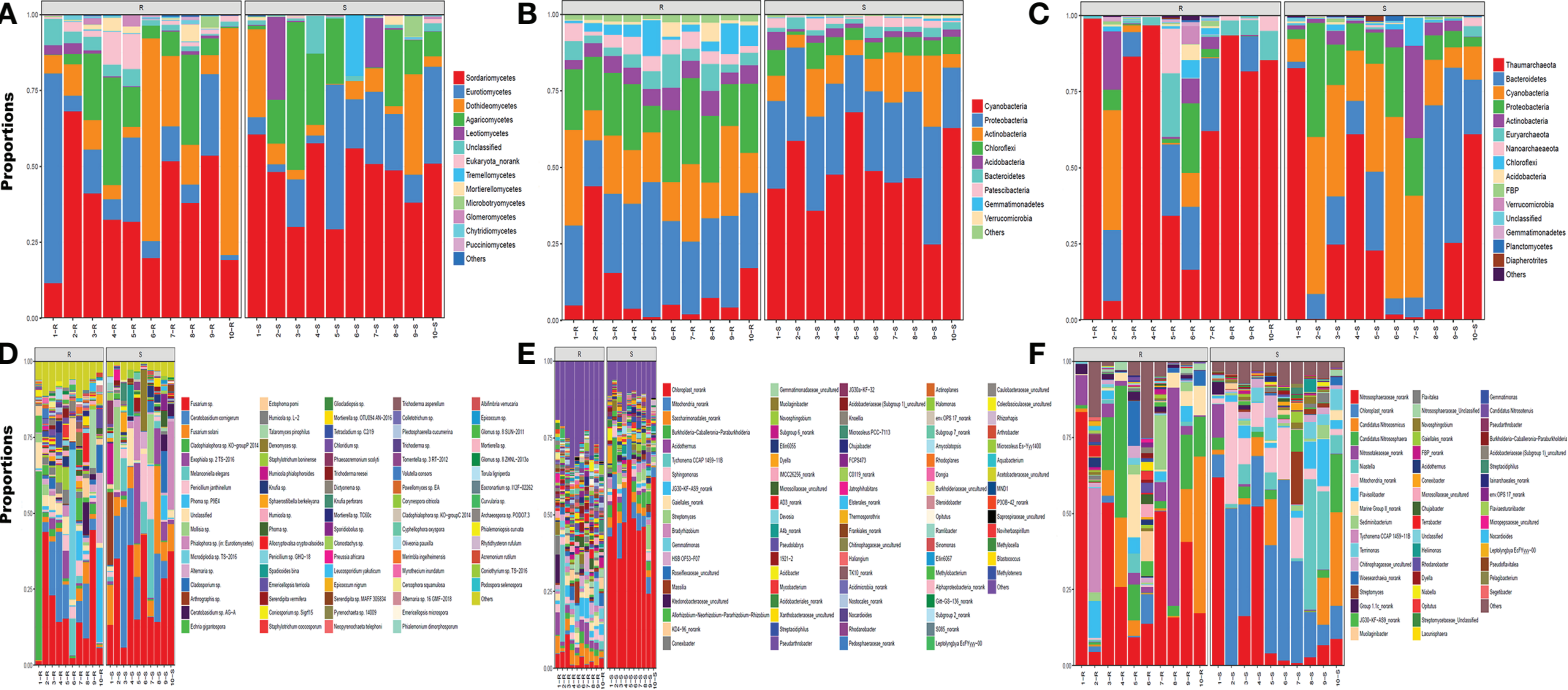
Soil and root microbial community composition of different biochar amendments in pot tests Notes: p-1 to p-10: CK, 1.5% C, 3% C, 4.5% C, 6%C, BF, BF+1.5% C, BF+3% C, BF+4.5% C and BF+6% C. S: soil; R: root. **(A–C)** representing the phylum and **(D–F)** representing the Genera. **(A, D)** showing the fungal, **(B, E)** showing bacterial and **(C, F)** showing the archaeal diversity. (clear pictures are provided in **Supplementary Material A–F**: **Figures S1–6**).

After biochar amendments, the soil and rhizosphere microbial communities changed. The fungi were mainly from the phyla Ascomycota, Basidiomycota, Chytridiomycota, Mucoromycota and some unclassified groups. In the classification of genera, Alternaria, Cladophialophora, Ceratobasidium, Cladosporium, Exophiala, Fusarium, Humicola, Penicillium, Phialophora, Phoma, and Stepylotrichum were dominant. Fusarium and Ceratobasidium were the most dominant genera whether base fertilizer was added or not. Cladophialophora was the dominant genus in the roots of the control group, and Arthrographis was the dominant genus in the soil (**Figures 2A, D**).

The main bacteria were in the phyla of Cyanobacteria, Acidobacteria, Actinobacteria, Proteobacteria, Patescibacteria, Gemmatimonadetes, Chloroflex and Bacteroidetes. The dominant genera in roots in the control group were Acidothermus, Conexibaber, and Ktedonobacteraceae_uncultured. The dominant genera in the soil were Acidibacter and Dyella. Burkholderia-Caballeronia-Paraburkholderia, Chloroplast_norank, Gemmatimonas, Nocardioides, Nostocales_norank, Roseiflexaceae_uncultured and Subgroup 6_norank were the dominant genera of the soil and root systems after biochar amendments (**Figures 2B, E**).

The main archaea were in the phyla of Bacteroidetes, Cyanobacteria, Proteobacteria and Thaumarchaeota. The dominant genera in the control group were Group 1.1c_norank, Nitrososphaeraceae_norank, and Nitrosotaleaceae_norank. After biochar amendments, Niastella was the dominant genus in the roots and Mitochondria_norank, Flavisolibacter, Chloroplast_norank, and Chitinophagaceae_uncultured were the dominant genera in the soil. The results showed that biochar could significantly change the microbial diversity of “Shatangju” roots and soil around the rhizosphere (**Figures 2C, F**).

### 3.3 Effects of different biochar amendments on “Shatangju” in the orchard

#### 3.3.1 Effects of different biochar amendments on the nutrient content of “Shatangju” leaves

The nutrient contents of leaves in the control group were as follows: total nitrogen 27.57 g/kg, total phosphorus 1.30 g/kg, total potassium 9.43 g/kg, calcium 26.14 g/kg, magnesium 2.80 g/kg, zinc 34.95 mg/kg, and boron 59.29 mg/kg. As shown in **Table 6**, after biochar amendments, the zinc content in leaves increased significantly, while the content of the other elements decreased.

**Table 6:** Changes in leaf nutrient contents with different amounts of biochar amendments in orchards.

Treatments	N	P	K	Ca	Mg	Zn	B
Control	27.57±0.12a	1.30±0.01a	9.43±0.03a	26.14±0.04ab	2.80±0.33a	34.95±0.10d	59.20±0.09b
C-1.2kg	27.49±0.09a	1.23±0.02ab	8.95±0.05a	25.07±0.07c	2.45±0.01a	73.81±0.16b	44.87±0.09e
C-2.4kg	25.86±0.05bc	1.14±0.01b	8.26±0.24b	23.08±0.08d	2.31±0.01b	74.08±0.08b	43.60±0.06f
C-3.6kg	25.84±0.09bc	1.18±0.01ab	7.42±0.37c	25.98±0.13b	2.57±0.07a	75.86±0.06a	48.69±0.05d
C-4.8kg	25.51±0.20c	1.20±0.10ab	8.00±0.11bc	25.21±0.10c	2.76±0.06a	74.12±0.02b	56.74±0.05c
C-6.0kg	26.25±0.25b	1.21±0.03ab	8.03±0.03bc	26.38±0.13a	2.65±0.04a	72.41±0.10c	61.83±0.09a

for N, P, K ,Ca and Mg, g/kg; for Zn and B, mg/kg. The lower case letters behind the values of the same indicator in each column are different, indicating that there is a significant difference between them(P<0.05).

#### 3.3.2 Effects of different biochar amendments on soil physical and chemical properties in the orchard

As shown in **Table 7**, biochar can significantly reduce the soil bulk density and increase the soil water content, field capacity and capillary porosity. With the increase in the amount of biochar amendments, the soil water content increased by 12.74%, 25.84%, 32.07%, 40.93% and 47.31%, the field water capacity increased by 17.28%, 28.10%, 39.15%, 53.93% and 67.08%, the capillary porosity increased by 8.75%, 11.42%, 14.10%, 19.56% and 23.61%, and the bulk density decreased by 7.55%, 13.21%, 18.22%, 22.56% and 26.12%. The results showed that biochar amendments could loosen the soil and improve the soil water retention property, so they could be used as an important measure to reduce the viscosity barrier of red soil. Biochar significantly increased the soil pH value and organic carbon content. Compared with that of the control, the soil organic matter in the treatment groups increased by 93.76%, 151.99%, 201.53%, 254.21% and 465.24%. This result indicated that biochar had obvious effects on improving soil acidification and fertilizer. The content of soil mineral elements in the biochar treatment groups was higher than that in the control group, and the changing trend was as follows: alkali hydrolyzable nitrogen and available phosphorus increased, and other elements increased first and then decreased. The inflection points of available potassium and boron were 2.4 kg/plant biochar, and the inflection points of exchangeable calcium, available magnesium and available zinc were 3.6 kg/plant biochar (**Table 8**).

**Table 7:** Changes in soil physical properties with different biochar amendments in orchards.

Treatments	Moisture content (%)	Field capacity (%)	Bulk density(g.cm^-1^)	Porosity (%)
CK	19.90±0.90d	30.00±0.10f	1.22±0.02a	3.65±0.03d
C-1.2kg	22.44±0.32c	35.18±0.01e	1.13±0.06a	3.97±0.08c
C-2.4kg	25.04±0.52b	38.43±0.16d	1.06±0.01a	4.01±0.01bc
C-3.6kg	26.28±0.33b	41.75±0.39c	1.00±0.30a	4.16±0.05b
C-4.8kg	28.05±0.21a	46.18±0.09b	0.94±0.04a	4.36±0.03a
C-6.0kg	29.31±0.35a	50.13±0.02a	0.90±0.09a	4.51±0.04a

The lower case letters behind the values of the same indicator in each column are different, indicating that there is a significant difference between them(P<0.05).

**Table 8:** Changes in soil chemical properties with different biochar amendments in orchards.

Treatments	pH	OMC	CEC	A-N	A-P	K	E-Ca	E-Mg	Zn	B
CK	4.80±0.02d	21.40±0.09f	08.04±0.04d	110.52±1.16e	22.71±0.10f	153.00±3.00c	889.45±9.21d	49.90±0.40f	3.88±0.04e	0.86±0.01c
C-1.2kg	6.54±0.03c	41.62±0.04e	09.85±0.04b	120.60±1.19d	39.81±0.06e	373.17±3.81b	1694.72±88.87c	94.40±0.40e	6.14±0.04b	1.00±0.10bc
C-2.4kg	6.86±0.12b	54.13±0.03d	10.08±0.03a	124.60±0.95c	47.09±0.04d	399.61±9.23a	2821.31±17.17a	135.56±0.56a	6.73±0.12a	1.00±0.00bc
C-3.6kg	7.13±0.08a	64.77±0.07c	09.37±0.04c	130.43±0.79b	53.65±0.08c	401.23±1.11a	2811.50±11.30a	131.64±0.54b	6.67±0.16a	1.46±0.03a
C-4.8kg	7.27±0.02a	76.08±0.08b	09.28±0.04c	132.53±0.91ab	55.73±0.05b	366.69±3.91b	2753.18±17.48ab	125.27±0.17c	5.68±0.14c	1.15±0.04b
C-6.0kg	7.31±0.01a	121.41±0.05a	09.32±0.02c	134.17±0.66a	58.61±0.01a	360.76±2.20b	2659.76±49.24b	114.10±0.21d	5.10±0.10d	0.96±0.05c

OMC, Organic matter content; CEC, cation exchange capacity; A, Available; E, Exchangeable; for exchangeable Ca, Mg and Mn, g/kg; for available N, P, K, Zn and B, mg/kg. The lower case letters behind the values of the same indicator in each column are different, indicating that there is a significant difference between them(P<0.05).

#### 3.3.3 Effects of different biochar amendments on soil and root microbial diversity in orchards

**Figure 3:**
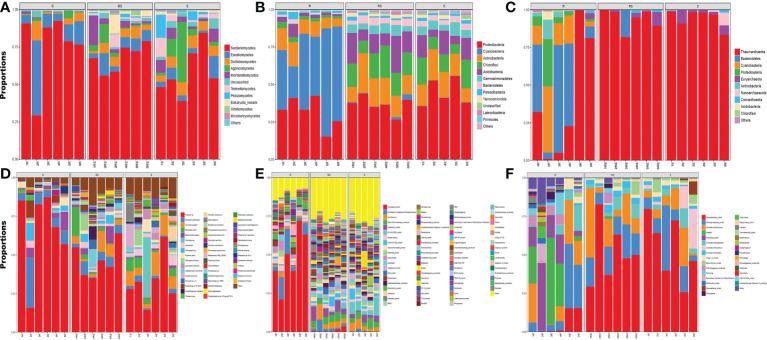
Soil and root microbial community composition of different biochar amendments in orchard Notes: p-1 to p-10: CK, 1.5% C, 3% C, 4.5% C, 6%C, BF, BF+1.5% C, BF+3% C, BF+4.5% C and BF+6% C. S: soil; R: root. **(A–C)** representing the phylum and **(D–F)** representing the Genera. **(A, D)** showing the fungal, **(B, E)** showing bacterial and **(C, F)** showing the archaeal diversity. (clear pictures are provided in **Supplementary Material A–F**: **Figures S7–12**).

The fungi were mainly in the phyla of Ascomycota, Basidiomycota, Mucoromycota and unclassified. The dominant genera were Arthrographis, Fusarium, Humicola, Lophistoma, Melanconiella, Mortierella, Penicillium, Phoma and Trichoderma. Fusarium, Humicola, Mortierella, and Penicillium were the most dominant genera in the control, and Arthrographis, Fusarium, Humicola, Mortierella and Penicillium were the most dominant genera in the root and rhizosphere soils. After different biochar amendments, the most dominant genera in root and rhizosphere soil changed into Arthrographis, Fusarium, Humicola, Lophistoma, Melanconiella, Mortierella, Penicillium, and Arthrographis in the root soil, and Fusarium, Humicola, Mortierella, Penicillium and Trechispora in the rhizosphere soil (**Figures 3A, D**).

The main bacteria phyla were Acidobacteria, Actinobacteria, Bacteroidetes, Chloroflexi, Cyanobacteria, Gemmatimonadetes and Proteobacteria; the dominant genera were Acidothermus, Burkholderia-Caballeronia-Paraburkholderia, Chloroplast_norank, Gemmatimonadaceae_uncultured, KF-JG30-C25_norank and Mitochondria_norank. Among them, the most dominant genera in the roots of the control group were Acidothermus, Bryobacter, Caldilineaceae_uncultured, Chitinophagaceae_uncultured, Chloroplast_norank and JG30-KF-AS9_norank. The dominant genera in rhizosphere soil were Acidothermus, Bryobacter, Chitinophagaceae_uncultured and KF-JG30-C25_norank. After biochar amendments, the most dominant genera in the roots were Acidothermus, Bryobacter, Burkholderia-Caballeronia-Paraburkholderia, Chloroplast_norank and Mitochondria_norank. The dominant genera in the rhizosphere soil were Acidothermus, Bryobacter, Burkholderia-Caballeronia-Paraburkholderia, Chitinophagaceae_uncultured, and KF-JG30-C25_norank (**Figures 3B, E**).

The main archaea were in the phyla of Bacteroidetes, Cyanobacteria, Euryarchaeota, Proteobacteria and Thaumarchaeota. Candidatus Nitrocosmicus, Candidatus Nitrososphaera, Candidatus Nitrosotalea, Candidatus Nitrosotenuis, Chitinophagaceae_uncultured, Chloroplast_norank, Group 1.1c_norank, Nitrososphaeraceae_norank, and Nitrosotaleaceae_norank were dominant genera. Candidatus Nitrocosmicus, FBP_norank and Flavitalea were the most dominant genera in the roots of the control group. Candidatus Nitrocosmicus was the most dominant genus in the rhizosphere soil. After different biochar amendments, the most dominant genera of roots were Candidatus Nitrososphaera, Candidatus Nitrocosmicus, Chloroplast_norank, Group 1.1c_norank, Marine Group II_norank, Nitrososphaeraceae_norank, and Nitrosotaleaceae_norank. In contrast, the most dominant genera in rhizosphere soil were Candidatus Nitrososphaera, Candidatus Nitrocosmicus, Group 1.1c_norank, Marine Group II_norank, Nitrososphaeraceae_norank, and Nitrosotaleaceae_norank (**Figures 3C, F**).

### 3.4 Effects of different biochar amendments on “Shatangju” fruit quality

After biochar amendments, the average fruit weight of “Shatangju” (37.44-40.44 g) did not change significantly, but the medium fruit proportion increased significantly. Compared with the control group, the number of medium fruits in each treatment group increased by 48.48%, 30.30%, 24.24%, 36.36% and 27.27%. Biochar amendments could reduce the peel thickness, and the peel thickness in groups with 3.6 kg/plant and 4.8 kg/plant biochar significantly decreased by 0.31 mm and 0.25 mm, respectively, compared with that of the control (2.06 mm), but it did not affect the fruit shape index (**Table 9**).

**Table 9:** Changes in physical fruit properties with different amounts of biochar amendment.

Treatments	Single fruitweight(g)	Proportion of middlefruits weight%	Proportion of middlefruits number%	Fruit shapeindex	Peel thick(mm)
Control	40.44±0.18b	52.62±0.10e	55.00±1.00e	0.78±0.01b	2.06±0.01a
C-1.2kg	39.83±0.25bc	79.97±0.19a	81.67±0.34a	0.82±0.00a	1.96±0.01bc
C-2.4kg	40.80±0.80b	69.49±0.16d	71.67±0.00c	0.80a±0.01b	1.75±0.01e
C-3.6kg	38.77±0.65cd	69.69±0.00d	68.33±1.00d	0.80±0.01ab	1.81±0.01d
C-4.8kg	42.89±0.31a	71.68±0.33c	75.00±0.50b	0.79±0.01ab	1.94±0.02c
C-6.0kg	37.44±0.10d	73.07±0.07b	70.00±0.00cd	0.80±0.01ab	2.00±0.02b

The lower case letters behind the values of the same indicator in each column are different, indicating that there is a significant difference between them(P<0.05).

The color value (de) and d (a/b) of the fruit color increased but did not reach a significant level compared with the control. The fruit surface brightness (dl) was the highest when 1.2 kg and 2.4 kg of biochar were added to each plant. The redness value (da) was highest at 6 kg of biochar per plant treatment. The yellow degree value (db) of each treatment was higher than that of the control, and the difference was significant (**Table 10**).

**Table 10:** Changes of citrus peel visual with different biochar amendments.

Treatments	de	dl-	da+	db+	da/b
Control	74.57±0.07a	33.48±0.13b	27.85±0.07c	60.18±0.08e	0.46±0.02a
C-1.2kg	76.12±0.11a	32.25±0.11c	28.88±0.20b	62.46±0.11a	0.46±0.00a
C-2.4kg	75.55±0.97a	33.89±0.09a	28.97±0.18b	60.66±0.12d	0.48±0.02a
C-3.6kg	75.99±0.11a	34.25±0.10a	28.88±0.08b	61.12±0.13c	0.47±0.01a
C-4.8kg	75.78±0.52a	32.42±0.13c	28.22±0.17c	62.06±0.10b	0.46±0.01a
C-6.0kg	75.89±0.24a	34.09±0.09a	29.86±0.18a	60.34±0.04de	0.49±0.01a

The lower case letters behind the values of the same indicator in each column are different, indicating that there is a significant difference between them(P<0.05).

**Table 11** shows that biochar can increase the soluble solids content/Titratable acid (SSC/TA), total sugar, reducing sugar, edible rate, and water content of “Shatangju”, and reduce the content of Titratable acid (TA). It can also increase the soluble solids content(SSC) content, vitamin C, and juice yield, except for individual treatments. When the biochar amendments were 2.4 kg/plant and 3.6 kg/plant, the fruit quality was better than that under the other treatments. Compared with that of the control, the SSC content increased by 4.96% and 5.79%, TA decreased by 16.67% and 8.33%, soluble solids content/Titratable acid (SSC/TA) increased by 24.48% and 15.24%, vitamin C content increased by 13.30% and 18.18%, total sugar increased by 11.36% and 13.21%, reducing sugar increased by 7.37% and 22.35%, edible rate increased by 2.96% and 3.79%, juice yield increased by 8.96% and 5.86%, and water content increased by 0.52% and 0.63%. These results supported that proper biochar amendments had an obvious effect on improving fruit quality.

**Table 11:** Changes in citrus fruit nutritional properties with different biochar amendments.

Treatments	SSC/%	TA/%	SSC/TA	Vitamin C(mg/100g)	Total sugar content /%	Reducing sugar content /%	Edible rate/%	Juice yield/%	Moisture content/%
Control	12.1±0.00c	0.48±0.01a	25.22±0.53d	21.43±0.03d	7.04±0.36c	4.34±0.04d	74.23±0.33c	51.92±0.70d	82.5±0.50a
C-1.2kg	12.5±0.10ab	0.43±0.00bc	29.07±0.23bc	22.98±0.46c	8.43±0.11ab	4.86±0.06b	75.17±0.10bc	50.19±0.10e	82.86±0.06a
C-2.4kg	12.7±0.10a	0.40±0.01d	31.78±1.04a	24.28±0.20b	7.84±0.25b	4.66±0.10bc	76.43±0.40ab	56.57±0.21b	82.93±0.58a
C-3.6kg	12.8±0.20a	0.44±0.01b	29.10±0.21b	25.33±0.30a	7.97±0.07ab	5.31±0.00a	77.04±0.52a	54.96±0.40c	83.02±0.02a
C-4.8kg	12.1±0.00c	0.44±0.00b	27.50±0.00c	23.74±0.20bc	8.43±0.03ab	4.75±0.05b	75.61±0.60bc	55.53±0.18bc	83.47±0.20a
C-6.0kg	12.2±0.00bc	0.41±0.01cd	29.77±0.73b	20.59±0.12d	8.61±0.07a	4.56±0.05c	76.28±0.32ab	58.25±0.27a	83.11±0.10a

de, the color of the peel; dl-, brightness of the peel; da+, redness of the peel; db+, yellowness of the peel; da/b, the ratio of Da and Db; SSC, soluble solids content; TA, Titratable acid. The lower case letters behind the values of the same indicator in each column are different, indicating that there is a significant difference between them(P<0.05).

## 4 Discussion

In this experiment, the organic waste of an orchard was fully utilized to prepare biochar. Organic waste from orchards includes “Shatangju” branches from pruning twice a year and grass from mowing five times a year. A total of 1187 kg per mu produced 437 kg of biochar per mu. The simple biochar furnace that we developed and produced can handle the preparation of biochar and the reuse of organic waste in the “Shatangju” orchard. With obvious effects, the biochar output can meet the orchard requirements and support the organic carbon cycle in the orchard.

Biochar is a kind of stable, insoluble and aromatic solid substance produced by the pyrolysis of agricultural solid wastes such as straw, rice husk, bamboo, wood and animal manure under at high temperature and anaerobic conditions (Tan et al., 2022). This study analyzed the effects of “Shatangju” branch biochar amendments on soil physical and chemical properties, soil and root microorganisms, leaf nutrition, and fruit quality of “Shatangju”(**Tables 4**–**11**). After biochar amendments, the soil bulk density decreased, and the soil water content, field capacity and capillary porosity increased significantly, consistent with previous research results (Wang et al., 2012a; Zeng et al., 2013; Li et al., 2014; Zhan et al., 2015). The physical properties of biochar determine these benefits. As a kind of carbon-rich microporous material, biochar has a large specific surface area, making its density far less than that of soil (Spokas et al., 2009). Biochar has a strong adsorption capacity and thus promotes the formation of soil aggregates, and its strong hydrophilicity can increase capillary porosity and improve soil water holding capacity (Downie et al., 2009). Biochar is an effective soil amendment (Glaser, 1998).

According to the sequencing analysis of soil and root microorganisms (**Figures 2**, **3**), biochar amendments significantly increased the number of species and the quantity of soil and root microorganisms in pot and field tests. Other dominant genera replaced many dominant genera in the control after biochar amendments. In the pot experiment, the abundance of beneficial bacteria such as Fusarium, Ceratobasidium, Chloroplast_norank, Mitochondria_norank and Gemmatimonas was the highest, and the content of harmful bacteria such as Cladophialophora was reduced when 3% biochar was added in the appropriate treatment. This is beneficial in degrading soil pollutants such as nitrogen oxides and heavy metals, increasing organic matter in mineral soil and fixing atmospheric nitrogen. The orchard environment is much more complex than the pot experiment environment with rain protection measures. The relative abundance of beneficial bacteria such as Fusarium, Humicola, Chloroplast_norank and Nitrososphaera was high, and the species of microbial colonies were more abundant in orchard soil. The high stability of biochar, which is a suitable addition amount, can prolong its retention in soil, improve soil structure, and affect the diversity of soil and root microbial communities, although orchard soil has a large volume and strong buffering capacity. Biochar amendments increased the soil microbial biomass in the peach orchard, especially the utilization rate of microbial carbon sources. However, high carbon is unfavorable to microbial diversity (Lu et al., 2020). The pH value of potato rhizosphere soil is important for soil microbial biomass, followed by organic carbon and total nitrogen (Xu et al., 2020). Biochar has a large specific surface area and nonvolatile ash, providing a more suitable niche for soil microorganisms. Biochar directly affected the soil pH value and increased the soil organic carbon content. We speculated that soil pH stability is the main reason for the increase microbial diversity in the soil and rhizosphere of “Shatangju” in the orchard and pot tests based on the results reported by Zhalnina et al. (2015) and Shi et al. (2021).

Relevant studies have suggested that biochar can also improve soil fertility, reduce nutrient fixation and leaching, promote nutrient absorption by plants, increase fertilizer utilization rate, and improve growth and development of most crops, while avoiding the disadvantages caused by the lime (Magrini-Bair et al., 2009; Laird et al., 2010; Wang et al., 2012a; Chen et al., 2013; Zhang et al., 2013a). This experiment also proved that biochar has these advantages in the “Shatangju” orchard and pot tests (**Tables 5**, **8**).

Biochar also exerts effects on improving the nutrient absorption of crops. Studies have shown that biochar amendments can improve the crop’s capacity to absorb nitrogen, phosphorus, potassium and other nutrients, and the absorption rate increases with the number of biochar amendments, however, when it exceeds a certain amount, it inhibits the absorption of nutrients by crops (Chan et al., 2007; Li et al., 2014; Khan et al., 2020). Our study showed that the contents of nitrogen, phosphorus, potassium, calcium, magnesium and boron in the leaves of “Shatangju” after biochar amendments were slightly lower than those of the control (**Tables 4**,**6**), consistent with the results of (Zhang et al., 2013c). It may be that biochar amendments can improve the soil nutrient efficiency and then improve the nutrient absorption of crops. However, the increase in plant biomass and fruit amount, carbohydrates produced by photosynthesis, and the transfer of nutrients lead to the dilution of leaf nutrients and the reduction of element contents. Meanwhile, biochar increased the content of available phosphorus, potassium, calcium and magnesium and other mineral elements in the soil and increased the absorption of these elements.

Previous studies have demonstrated that (Fang et al., 2014; Zhang et al., 2014) biochar amendments can increase the aboveground dry matter accumulation and crop yield, but the effect on crop quality is rarely mentioned. The present experiment also proved the effect of biochar in the production of “Shatangju”. The different proportions of biochar can improve “Shatangju” internal and external qualities to varying degrees, especially the proportion of medium fruit. The price of medium fruit of “Shatangju” is high so that biochar amendments can increase the economic benefits after harvest for the grower (**Tables 9**–**11**).

In this experiment, biochar amendments in pot and field tests significantly improved the soil pH value and organic matter contents (OMC), hydrolyzable alkali nitrogen, phosphorus, potassium, exchangeable calcium, exchangeable magnesium, zinc, available boron and CEC. Many pot tests have found similar findings and analyzed the related reasons (Kimetu and Lehmann, 2010; Gao et al., 2012; Han et al., 2012; Zhang et al., 2013a; Zhang et al., 2013c; Zhao et al., 2015; Zhu et al., 2015). However, there is not a complete design for the optimal amount of biochar. It is generally believed that the effect of biochar on crop growth, yield and quality is closely related to the biochar amount and soil properties (Khan et al., 2021a). Asai et al. (2009) found that rice yield increased with biochar amendments, but when biochar amendments reached 16 t/hm^2^, rice yield did not increase due to nitrogen deficiency. In a pot test on sandy loam, Vaccari et al. (2011) showed that the biomass of ryegrass increased by 20% and 52% when biochar amendments were 30 t/hm^2^ and 60 t/hm^2^, respectively, but decreased when biochar amendments were 100 t/hm^2^ and 200 t/hm^2^ compared with that of the control group. This study found that in the pure soil test, whether or not chemical fertilizer is applied, 3% biochar amendments can provide a suitable pH value for “Shatangju” growth and are relatively stable. Due to the limited amount of planting soil in the pot test, whether or not fertilizer is applied, 1.5%-3% biochar can improve the soil. In the field test, the biochar at 2.4-3.6 kg/plant (approximately 4% - 6% of the mixed soil sample, namely, approximately it is about 4% - 6% of the soil in the fertilizing ditch 80 cm long, 30 cm wide and 30 cm deep) is suitable for the growth and development of “Shatangju” with proper soil pH value, organic matter content (OMC), large, medium and trace element content and leaf element content, and improved fruit quality. Therefore, the amount of biochar added in the open environment (if the garden) can be slightly adjusted according to the results of the closed environment test (pure soil test and pot test). Of course, the amount of biochar used in the field needs to be determined by the soil, variety, tree age and planting density, and it should be noted that the effect of not always better the amount is.

## 5 Conclusion

A simple small-scale biochar furnace is beneficial for the “Shatangju” orchard. It can convert the carbon fixed by “Shatangju” plants and orchard grasses into biochar and change the physical and chemical properties of soil and microbial colonies in the soil by applying 2.4-3.6 kg biochar per plant, thus affecting the absorption and utilization of soil nutrients by “Shatangju” roots, directly increasing the yield and improving the quality of fruit. The results of the closed environment test (pure soil test and pot test) and the open environment test (if it is a garden) can refer to each other in terms of the added biochar in order to obtain a model of the organic carbon cycle in the orchard. It provides a method deal with the waste in the orchard, address the problem of biochar shortage, and maintain the cycle of organic carbon in the orchard, which provides a theoretical basis for the fertilization, soil improvement and rational agriculture of biochar in “Shatangju” orchards.

## Data availability statement

The original contributions presented in the study are publicly available. This data can be found here: NCBI, PRJNA889182.

## Author contributions

YG: methodology, data curation, visualization and investigation, formal analysis and writing the original draft preparation. BQ: data curation, fund acquisition, project administration and resources. ZK: formal analysis and data curation. HJ: visualization and investigation. QJ: supervision, conceptualization, resources, writing, reviewing and editing, project administration, and funding acquisition. MK: conceptualization, software, writing, reviewing, and editing. QF: software, formal analysis and resources. All authors contributed to the article and approved the submitted version.

## Funding

This study was supported by the Laboratory of Lingnan Modern Agriculture Project (NT2021003), China Agriculture Research System of MOF and MARA (CARS-26) and Guangdong Provincial Key Laboratory of Environmental Health and Land Resource (Grant No. 2020B121201014).

## Conflict of interest

The authors declare that the research was conducted in the absence of any commercial or financial relationships that could be construed as a potential conflict of interest.

## Publisher’s note

All claims expressed in this article are solely those of the authors and do not necessarily represent those of their affiliated organizations, or those of the publisher, the editors and the reviewers. Any product that may be evaluated in this article, or claim that may be made by its manufacturer, is not guaranteed or endorsed by the publisher.

## References

[B1] Ahmad M. Lee S. S. Lim J. E. Lee S. E. Cho J. S. Moon D. H.. (2014). Speciation and phytoavailability of lead and antimony in a small arms range soil amended with mussel shell, cow bone and biochar: EXAFS spectroscopy and chemical extractions. Chemosphere 95, 433–441. doi: 10.1016/j.chemosphere.2013.09.077 24183621

[B2] Ahmad M. Ok Y. S. Kim B. Y. Ahn J. H. Lee Y. H. Zhang M.. (2016). Impact of soybean stover- and pine needle-derived biochars on Pb and as mobility, microbial community, and carbon stability in a contaminated agricultural soil. J. Environ. Manage. 166, 131–139. doi: 10.1016/j.jenvman.2015.10.006 26496843

[B3] Asai H. Samson B. K. Stephan H. M. Songyikhangsuthor K. Homma K. Kiyono Y.. (2009). Biochar amendment techniques for upland rice production in northern laos. 1. soil physical properties, leaf SPAD and grain yield. F. Crop Res. 111, 81–84. doi: 10.1016/j.fcr.2008.10.008

[B4] Bao S. (2005). Soil agrochemical analysis. 3rd ed (Beijing: China Agricultural Publishing).

[B5] Bashir S. Rizwan M. S. Salam A. Fu Q. Zhu J. Shaaban M.. (2018). Cadmium immobilization potential of rice straw-derived biochar, zeolite and rock phosphate: Extraction techniques and adsorption mechanism. Bull. Environ. Contam. Toxicol. 100, 727–732. doi: 10.1007/s00128-018-2310-z 29516140

[B6] Brahmakshatriya R. D. Donker J. D. (1971). Five methods for determination of silage dry matter. J. Dairy Sci. 54, 1470–1474. doi: 10.3168/jds.S0022-0302(71)86049-6

[B7] Bruun E. W. Petersen C. T. Hansen E. Holm J. K. Hauggaard-Nielsen H. (2014). Biochar amendment to coarse sandy subsoil improves root growth and increases water retention. Soil Use Manage. 30, 109–118. doi: 10.1111/sum.12102

[B8] Chaisiri C. Liu X. Y. Yin W. X. Luo C. X. Lin Y. (2021). Morphology characterization, molecular phylogeny, and pathogenicity of diaporthe passifloricola on citrus reticulata cv. nanfengmiju in Jiangxi province, China. Plants 10, 1–20. doi: 10.3390/plants10020218 PMC7911537 33498730

[B9] Chan K. Y. Van Zwieten L. Meszaros I. Downie A. Joseph S. (2007). Agronomic values of greenwaste biochar as a soil amendment. Aust. J. Soil Res. 45, 629–634. doi: 10.1071/SR07109

[B10] Chen W. Zhang W. Meng J. (2013). Advances and prospects in research of biochar utilization in agriculture. Sci. Agric. Sin. 46, 3324–3333. doi: 10.3864/j.issn.0578-1752.2013.16.003

[B11] Downie A. Crosky A. Munroe P. (2009). “Physical properties of biochar,” in Biochar for environmental management, vol. 448 . Eds. Lehmann J. Joseph S. (London: Taylor & Francis). doi: 10.4324/9781849770552

[B12] Du Z-I. Ren T-s. Hu C-s. Zhang Q-z. Blanco-Canqui H. (2013). Soil aggregate stability and aggregate-associated carbon under different tillage systems in the North China Plain. J. Integr. Agric. 12, 2114–2123. doi: 10.1016/S2095-3119(13)60428-1

[B13] Fang B. Li X. Zhao B. Zhong L. (2014). Influence of biochar on soil physical and chemical properties and crop yields in rainfed field. Ecol. Environ. Sci. 23, 1292–1297. doi: 10.16258/j.cnki.1674-5906.2014.08.006

[B14] Gao H. He X. Chen X. Zhang W. Geng Z. (2012). Effect of biochar and biochar-based ammonium nitrate fertilizers on soil chemical properties and crop yield. J. Agro-Environment Sci. 31, 1948–1955.

[B15] Glaser B. (1998). Black carbon in soils: the use of benzene carboxylic acids as specific markers. Org. Geochem. 29, 811–819. doi: 10.1016/S0146-6380(98)00194-6

[B16] Han G. M. Meng J. Zhang W. M. Chen W. F. (2012). Effect of biochar on microorganisms quantity and soil physicochemical property in rhizosphere of spinach (Spinacia oleracea l.). J. Shenyang Agric. Univ. (Social Ed. 43, 515–520.

[B17] Iqbal A. Liang H. Mcbride S. G. Yuan P. Ali I. Zeeshan M.. (2022). Manure applications combined with chemical fertilizer improves soil functionality , microbial biomass and rice production in a paddy field. Agron. J. 114, 1–16. doi: 10.1002/agj2.20990

[B18] Jiang H. Guo Y. Guo L. Zhou X. Hu Y. Ji Q. (2015). Soil nutrient and leaf nutrient status of yellow shatang orange orchard in xijiang river basin of guangdong province. South China Fruits 43, 59–63. doi: 10.13938/j.issn.1007-1431.20140598

[B19] Kang C. Cao J. Sun J. Zheng G. Wang Y. Chen K.. (2022). Comparison of physiochemical characteristics of citrus reticulata cv. shatangju fruit with different fruit sizes after storage. Food Packag. Shelf Life 31, 100774. doi: 10.1016/j.fpsl.2021.100774

[B20] Khan Z. Khan M. N. Zhang K. Luo T. Zhu K. Hu L. (2021a). The application of biochar alleviated the adverse effects of drought on the growth, physiology, yield and quality of rapeseed through regulation of soil status and nutrients availability. Ind. Crops Prod. 171, 113878. doi: 10.1016/j.indcrop.2021.113878

[B21] Khan Z. Nauman Khan M. Luo T. Zhang K. Zhu K. Rana M. S.. (2021b). Compensation of high nitrogen toxicity and nitrogen deficiency with biochar amendment through enhancement of soil fertility and nitrogen use efficiency promoted rice growth and yield. GCB Bioenergy 13, 1765–1784. doi: 10.1111/gcbb.12884

[B22] Khan Z. Zhang K. Khan M. N. Bi J. Zhu K. Luo L.. (2022). How biochar affects nitrogen assimilation and dynamics by interacting soil and plant enzymatic activities: Quantitative assessment of 2 years potted study in a rapeseed-soil system. Front. Plant Sci. 13. doi: 10.3389/fpls.2022.853449 PMC8960854 35360339

[B23] Khan Z. Zhang K. Khan M. N. Fahad S. Xu Z. Hu L. (2020). Coupling of biochar with nitrogen supplements improve soil fertility, nitrogen utilization efficiency and rapeseed growth. Agronomy 10, 1661. doi: 10.3390/agronomy10111661

[B24] Kimetu J. M. Lehmann J. (2010). Stability and stabilisation of biochar and green manure in soil with different organic carbon contents. Aust. J. Soil Res. 48, 577–585. doi: 10.1071/SR10036

[B25] Laird D. A. Fleming P. Davis D. D. Horton R. Wang B. Karlen D. L. (2010). Impact of biochar amendments on the quality of a typical Midwestern agricultural soil. Geoderma 158, 443–449. doi: 10.1016/j.geoderma.2010.05.013

[B26] Lehmann J. Rillig M. C. Thies J. Masiello C. A. Hockaday W. C. Crowley D. (2011). Biochar effects on soil biota - a review. Soil Biol. Biochem. 43, 1812–1836. doi: 10.1016/j.soilbio.2011.04.022

[B27] Li H. (2000). Principles and techniques of plant physiological and biochemical experiments (Beijing: Higher Education Press).

[B28] Li T. Shi S. Goel S. Shen X. Xie X. Chen Z.. (2019). Recent advancements in mesoporous silica nanoparticles towards therapeutic applications for cancer. Acta Biomat. 89, 1–13. doi: 10.1016/j.actbio.2019.02.031 30797106

[B29] Liu B. Xia H. Jiang C. Riaz M. Yang L. Chen Y.. (2022). 14 year applications of chemical fertilizers and crop straw effects on soil labile organic carbon fractions, enzyme activities and microbial community in rice-wheat rotation of middle China. Sci. Tot. Environ. 841, 156608. doi: 10.1016/j.scitotenv.2022.156608 35700778

[B30] Li Q. Wu F. Li T. Su X. Jiang G. Qu H.. (2012). ). 1-methylcyclopropene extends the shelf-life of “Shatangju” mandarin (Citrus reticulate blanco) fruit with attached leaves. Postharvest Biol. Technol. 67, 92–95. doi: 10.1016/j.postharvbio.2012.01.001

[B31] Li X. Zhang J. Li L. Pan G. Zhang X. Zheng J.. (2014). Effects of biochar amendment on maize growth and soil properties in Huang-Huai-Hai plain. Soil 46, 269–274. doi: 10.13758/j.cnki.tr.2014.02.012

[B32] Lu H. Yan M. Hung M. Yin W. Wang Y. Wen X.. (2020). Effects of biochar on soil microbial community and functional genes of a land fi ll cover three years after ecological restoration. Sci. Tot. Environ. 717, 137133. doi: 10.1016/j.scitotenv.2020.137133 32062262

[B33] Magrini-Bair K. A. Czernik S. Pilath H. M. Evans R. J. Maness C. Leventhal J. (2009). Biomass derived, carbon sequestering, designed fertilizers. Ann. Environ. Sci. 3, 217–225.

[B34] Meng L. Sun T. Li M. Saleem M. Zhang Q. Wang C. (2019). Soil-applied biochar increases microbial diversity and wheat plant performance under herbicide fomesafen stress. Ecotoxicol. Environ. Saf. 171, 75–83. doi: 10.1016/j.ecoenv.2018.12.065 30597319

[B35] Mikula K. Izydorczyk G. Skrzypczak D. Mironiuk M. Moustakas K. Witek-Krowiak A. (2019). Controlled release micronutrient fertilizers for precision agriculture – A review. Sci. Total Environ. 712, 1–8. doi: 10.1016/j.scitotenv.2019.136365 31935544

[B36] Oleszczuk P. Jośko I. Futa B. Pasieczna-Patkowska S. Pałys E. Kraska P. (2014). Effect of pesticides on microorganisms, enzymatic activity and plant in biochar-amended soil. Geoderma 214–215, 10–18. doi: 10.1016/j.geoderma.2013.10.010

[B37] Oni B. A. Oziegbe O. Olawole O. O. (2019). Significance of biochar application to the environment and economy. Ann. Agric. Sci. 64, 222–236. doi: 10.1016/j.aoas.2019.12.006

[B38] Oshunsanya S. O. Aliku O. O. (2016). “Biochar technology for sustainable organic farming,” in Organic farming - a promising way of food production. Ed. Konvalina P. (London, United Kingdom: IntechOpen), 111–129. doi: 10.5772/61440

[B39] Pawlak-Kruczek H. Niedzwiecki L. Sieradzka M. Mlonka-Mędrala A. Baranowski M. Serafin-Tkaczuk M.. (2020). Hydrothermal carbonization of agricultural and municipal solid waste digestates – structure and energetic properties of the solid products. Fuel 275, 117837. doi: 10.1016/j.fuel.2020.117837

[B40] Puget P. Lal R. (2005). Soil organic carbon and nitrogen in a mollisol in central Ohio as affected by tillage and land use. Soil Tillage Res. 80, 201–213. doi: 10.1016/j.still.2004.03.018

[B41] Shi Y. Li Y. Yang T. Chu H. (2021). Threshold effects of soil pH on microbial co-occurrence structure in acidic and alkaline arable lands. Sci. Tot. Environ. 800, 149592. doi: 10.1016/j.scitotenv.2021.149592 34426307

[B42] Spokas K. A. Koskinen W. C. Baker J. M. Reicosky D. C. (2009). Chemosphere impacts of woodchip biochar additions on greenhouse gas production and sorption / degradation of two herbicides in a Minnesota soil. Chemosphere 77, 574–581. doi: 10.1016/j.chemosphere.2009.06.053 19647284

[B43] Tan S. Mathiyazhagan Narayanan D. T. T. H. Ito N. Unpaprom Y. Pugazhendhi A. Chi N. T. L.. (2022). A perspective on the interaction between biochar and soil microbes: A way to regain soil eminence. Environ. Int. 214, 113832. doi: 10.1016/j.envres.2022.113832 35810814

[B44] Tardy V. Spor A. Mathieu O. Lévèque J. Terrat S. Plassart P.. (2015). Shifts in microbial diversity through land use intensity as drivers of carbon mineralization in soil. Soil Biol. Biochem. 90, 204–213. doi: 10.1016/j.soilbio.2015.08.010

[B45] Tian S. Ning T. Wang Y. Liu Z. Li G. Li Z. (2016). Crop yield and soil carbon responses to tillage method changes in north China. Soil Tillage Res. 163, 207–213. doi: 10.1016/j.still.2016.06.005

[B46] Vaccari F. P. Baronti S. Lugato E. Genesio L. Castaldi S. Fornasier F.. (2011). Biochar as a strategy to sequester carbon and increase yield in durum wheat. Eur. J. Agron. 34, 231–238. doi: 10.1016/j.eja.2011.01.006

[B47] Wang Z. Li H. Lin J. Tian J. Wu Z. (2012b). Study on quality and storability of different size of shatangju fruits. Storage Process 12, 6–11.

[B48] Wang Y. Yuan Y. Liu S. (2009). Comparative study on determination methods of corn silage. China Dairy Cattle 2009, 14–17.

[B49] Wang D. Zhang X. Jiang C. Peng S. (2012a). Biochar research advances regarding soil improvement and crop response. Chin. J. Eco-Agricul0 20, 963–967. doi: 10.3724/SP.J.1011.2012.00963

[B50] Wu H. Zeng G. Liang J. Chen J. Xu J. Dai J.. (2016). Responses of bacterial community and functional marker genes of nitrogen cycling to biochar, compost and combined amendments in soil. Appl. Microbiol. Biotechnol. 100, 8583–8591. doi: 10.1007/s00253-016-7614-5 27338575

[B51] Xia H. Riaz M. Zhang M. Liu B. El-desouki Z. Jiang C. (2020). Biochar increases nitrogen use efficiency of maize by relieving aluminum toxicity and improving soil quality in acidic soil. Ecotoxicol. Environ. Saf. 196, 110531. doi: 10.1016/j.ecoenv.2020.110531 32244117

[B52] Xu Y. Ma S. Zhu B. Zhang X. Xing Y. Duan M.. (2020). Effects of the combined application of biochar and chemical fertilizer on fertility and microbial characteristics of purple soil and yield and quality of oilseed rape. Acta Prataculturae Sin. 29, 121–131. doi: 10.11686/cyyxb2019338

[B53] Yu H. Zou W. Chen J. Chen H. Yu Z. Huang J.. (2019). Biochar amendment improves crop production in problem soils: A review. J. Environ. Manage. 232, 8–21. doi: 10.1016/j.jenvman.2018.10.117 30466010

[B54] Zeng A. Liao Y. Zhang J. Sui Y. Wen X. (2013). Effects of biochar on soil moisture, organic carbon and available nutrient contents in manural loessial soils. J. Agro-Environment Sci. 32, 1009–1015. doi: 10.11654/jaes.2013.05.019

[B55] Zhalnina K. Dias R. de Quadros P. D. Davis-Richardson A. Camargo F. A. O. Clark I. M.. (2015). Soil pH determines microbial diversity and composition in the park grass experiment. Microb. Ecol. 69, 395–406. doi: 10.1007/s00248-014-0530-2 25395291

[B56] Zhang N. Li J. Liu X. Liu Y. Wang Y. Liang H.. (2014). Effects of biochar on growth and yield of summer maize. J. Agro-Environment Sci. 33, 1569–1574. doi: 10.11654/jaes.2014.08.015

[B57] Zhang X. Wang D. Jiang C. Peng S. (2013b). Biochar and research advances of biochar in acidic soil improvement. Hubei Agric. Sci. 52, 997–1000. doi: 10.14088/j.cnki.issn0439-8114.2013.05.037

[B58] Zhang X. Wang D. Jiang C.-C. Zhu P. Lei J. Peng S.-A. (2013a). Effect of biochar on physicochemical properties of red and yellow brown soils in the south China region. Chin. J. Eco-Agricul0 21, 979–984. doi: 10.3724/sp.j.1011.2013.00979

[B59] Zhang X. Wang D. Zhu P. Jiang C. Peng S. (2013c). Effects of biochar on improvement of acid red soil and growth of navel orange seedlings. South China Fruits 42, 38–41. doi: 10.13938/j.issn.1007-1431.2013.06.003

[B60] Zhan X. Peng J. Wang Y. Liu Y. Chen K. Han X.. (2015). Influences of application of biochar and biochar-based fertilizer on brown soil physiochemical properties and peanut yields. Plant Nutr. Fertil Sci. 21, 1633–1614. doi: 10.11674/zwyf.2015.0631

[B61] Zhao Q. Meng J. Chen W. (2015). Effect of biochar on growth of brassica campestris l. ssp. pekinesis (lour) olsson. J. Agro-Environment Sci. 34, 2394–2401.

[B62] Zhu P. Ying J. Peng S. Jiang C. (2015). Effects of biochar and lime on soil physicochemical properties and tobacco seedling growth in red soil. J. Agric. Resour. Environ. 32, 590–595.

