# Supplementary material for: The potential for biochar application in “Shatangju” (Citrus reticulate cv.) orchard on acid red soil: Biochar prepared from its organic waste in an orchard
Source: Front Plant Sci. 2022 Oct 20;13:1001740. doi: 10.3389/fpls.2022.1001740 (PMC9632651; doi:10.3389/fpls.2022.1001740)
Supplement: Supplementary file 1 [file DataSheet_1.pdf]

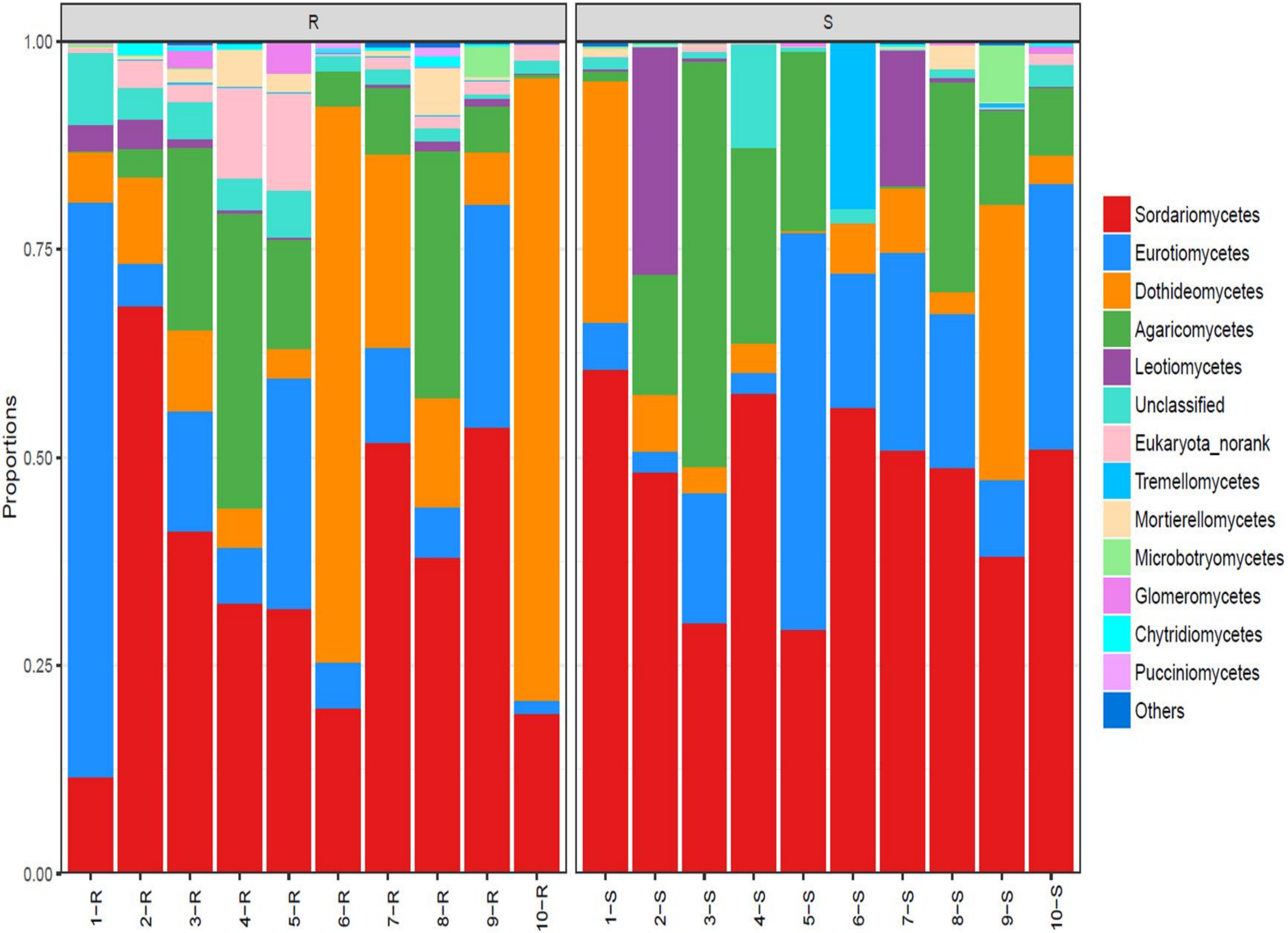

Figure S1: Phylum of soil and root fungal community composition of different biochar amendments in pot tests (Figure 2A)

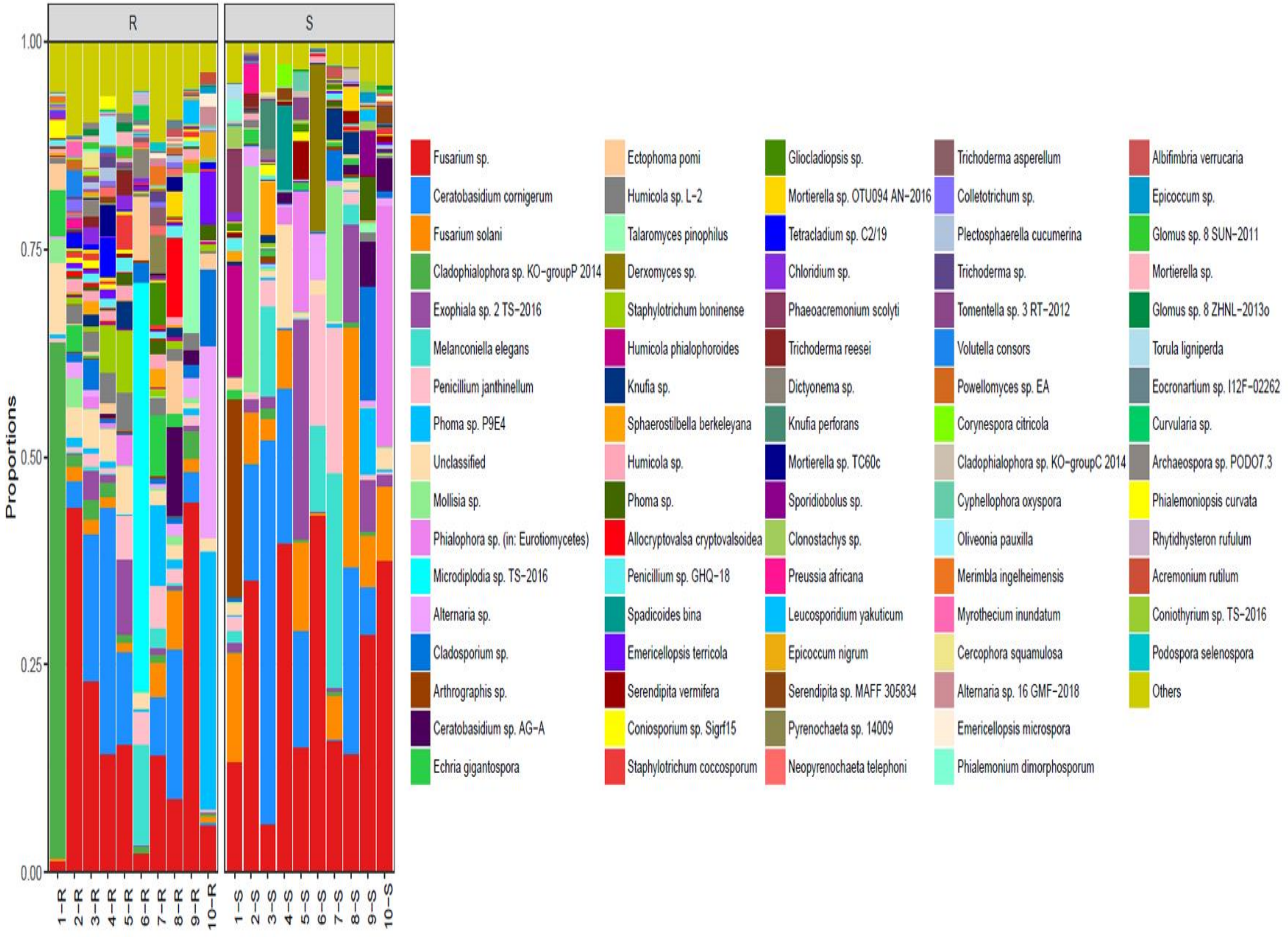

Figure S2: Genera of soil and root fungal community composition of different biochar amendments in pot tests (Figure 2D)

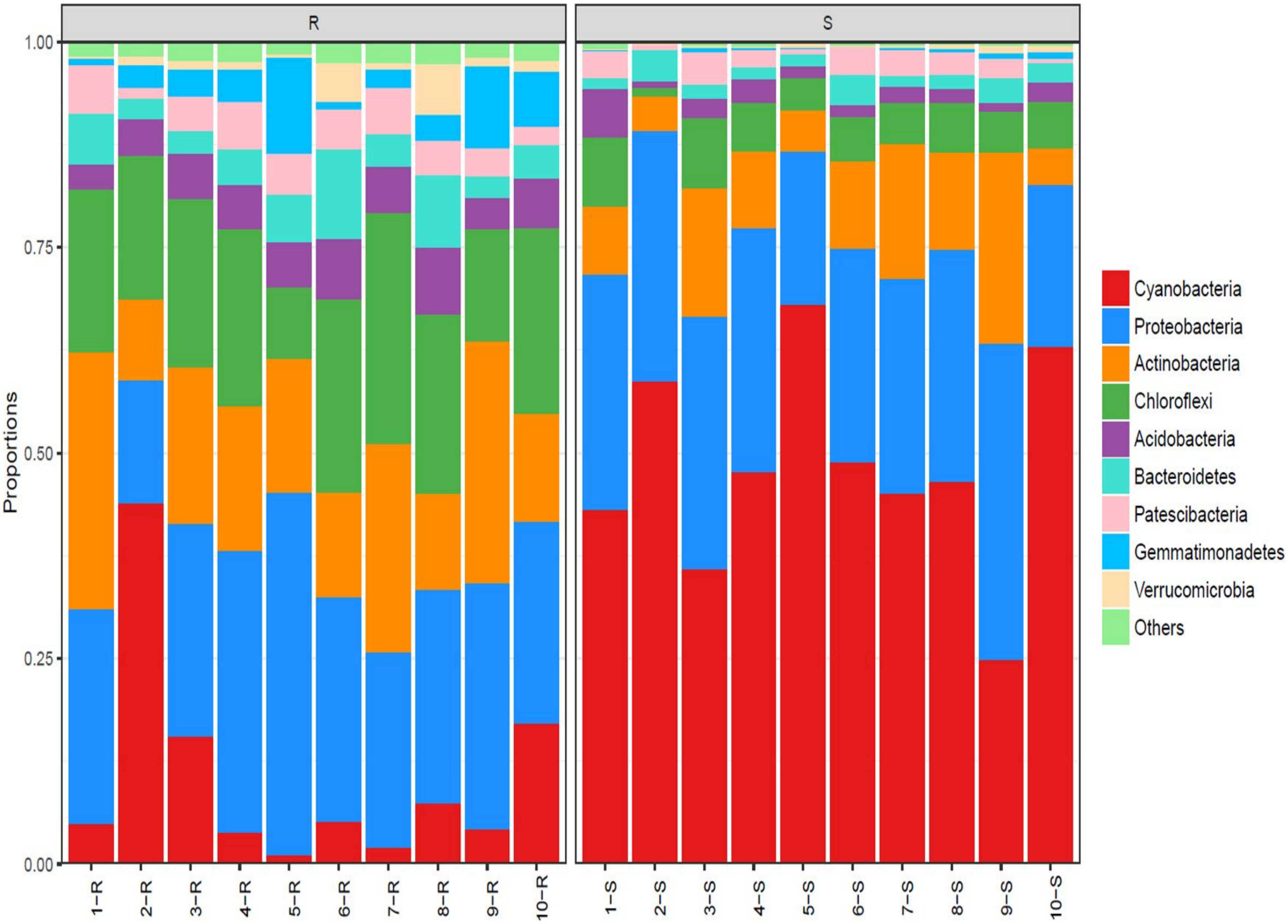

Figure S3: Phylum of soil and root bacterial community composition of different biochar amendments in pot tests (Figure 2B)

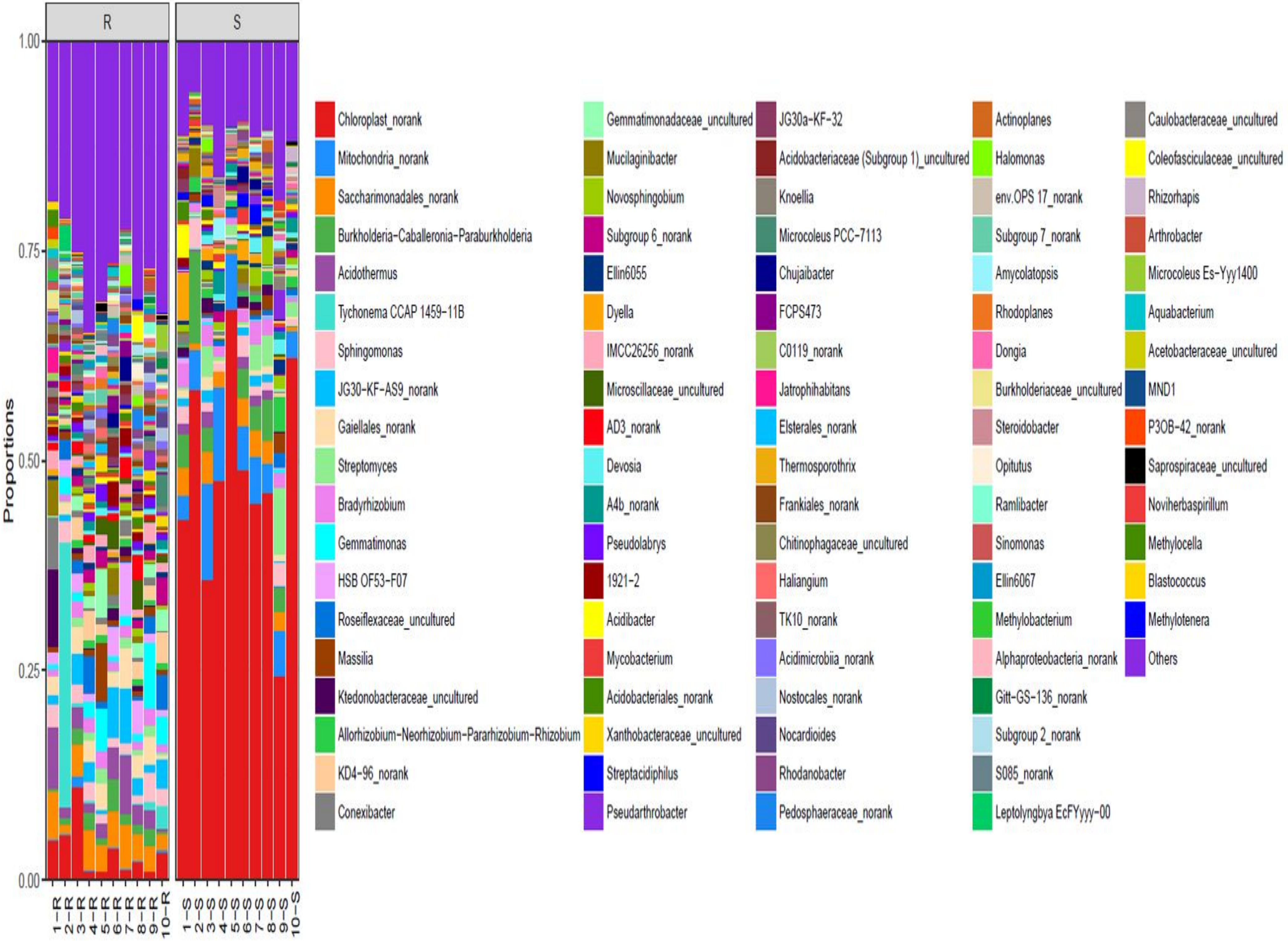

Figure S4: Genera of soil and root bacterial community composition of different biochar amendments in pot tests (Figure 2E)

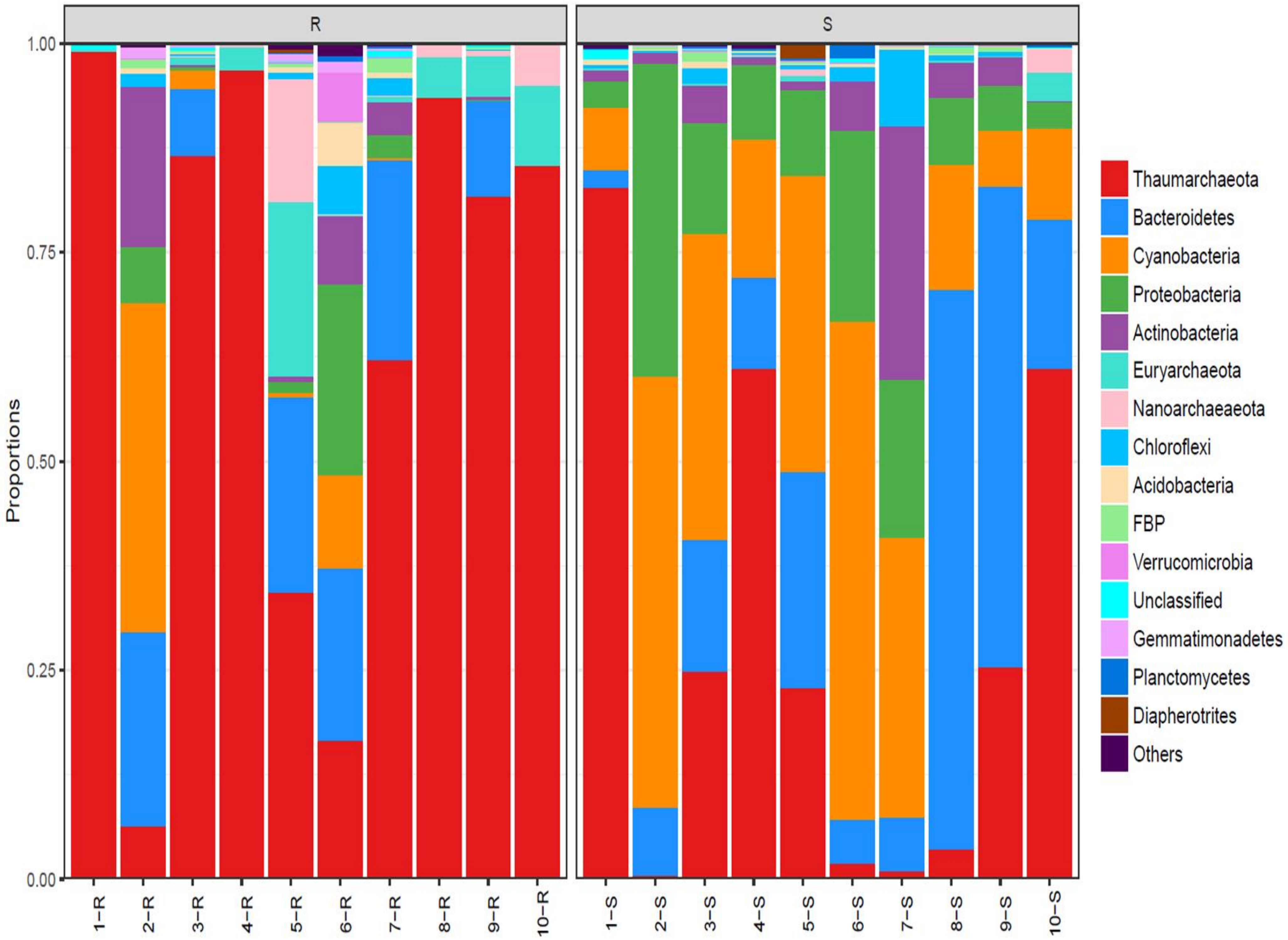

Figure S5: Phylum of soil and root Archaea community composition of different biochar amendments in pot tests (Figure 2C)

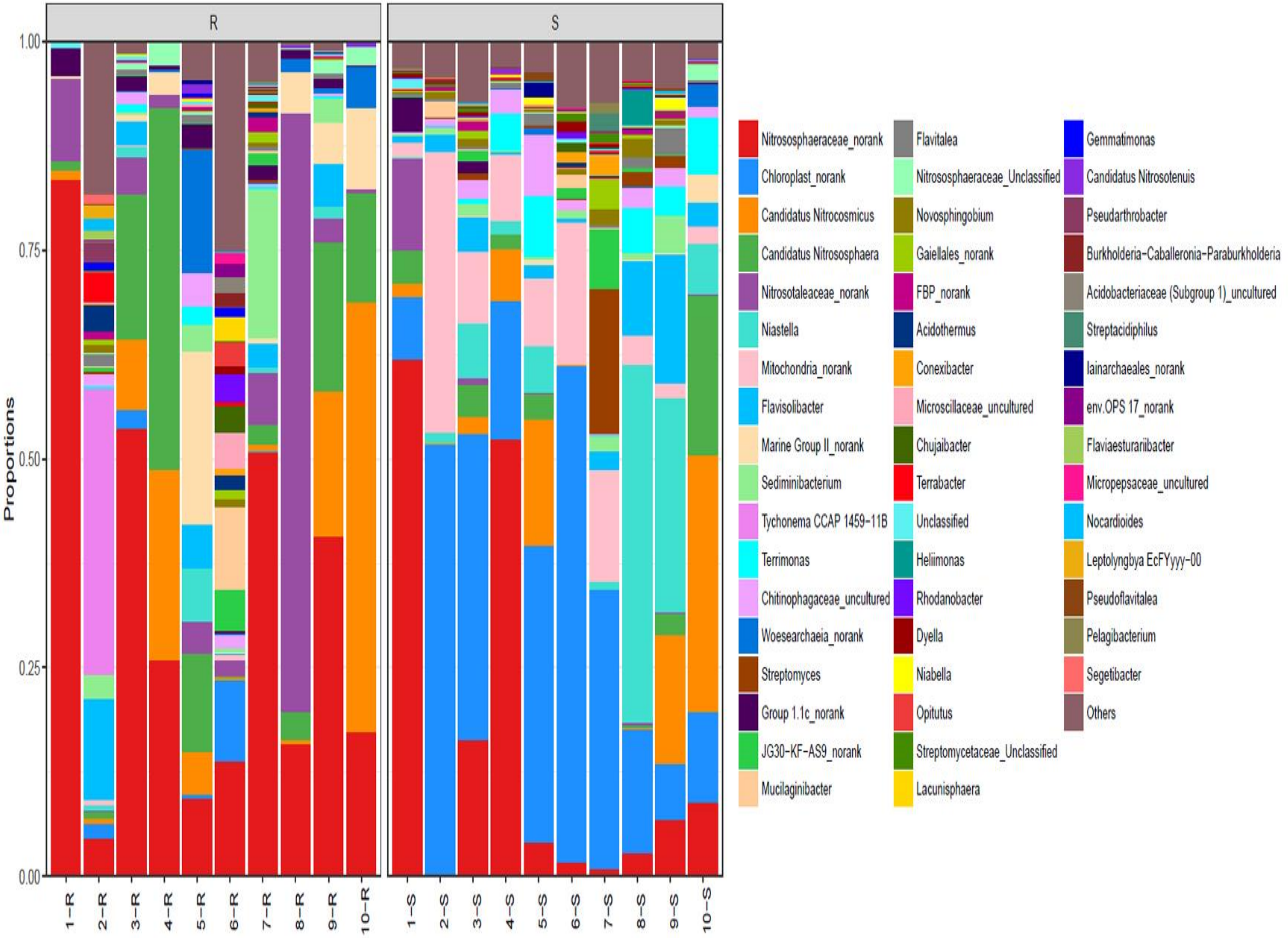

Figure S6: Genera of soil and root Archaea community composition of different biochar amendments in pot tests (Figure 2F)

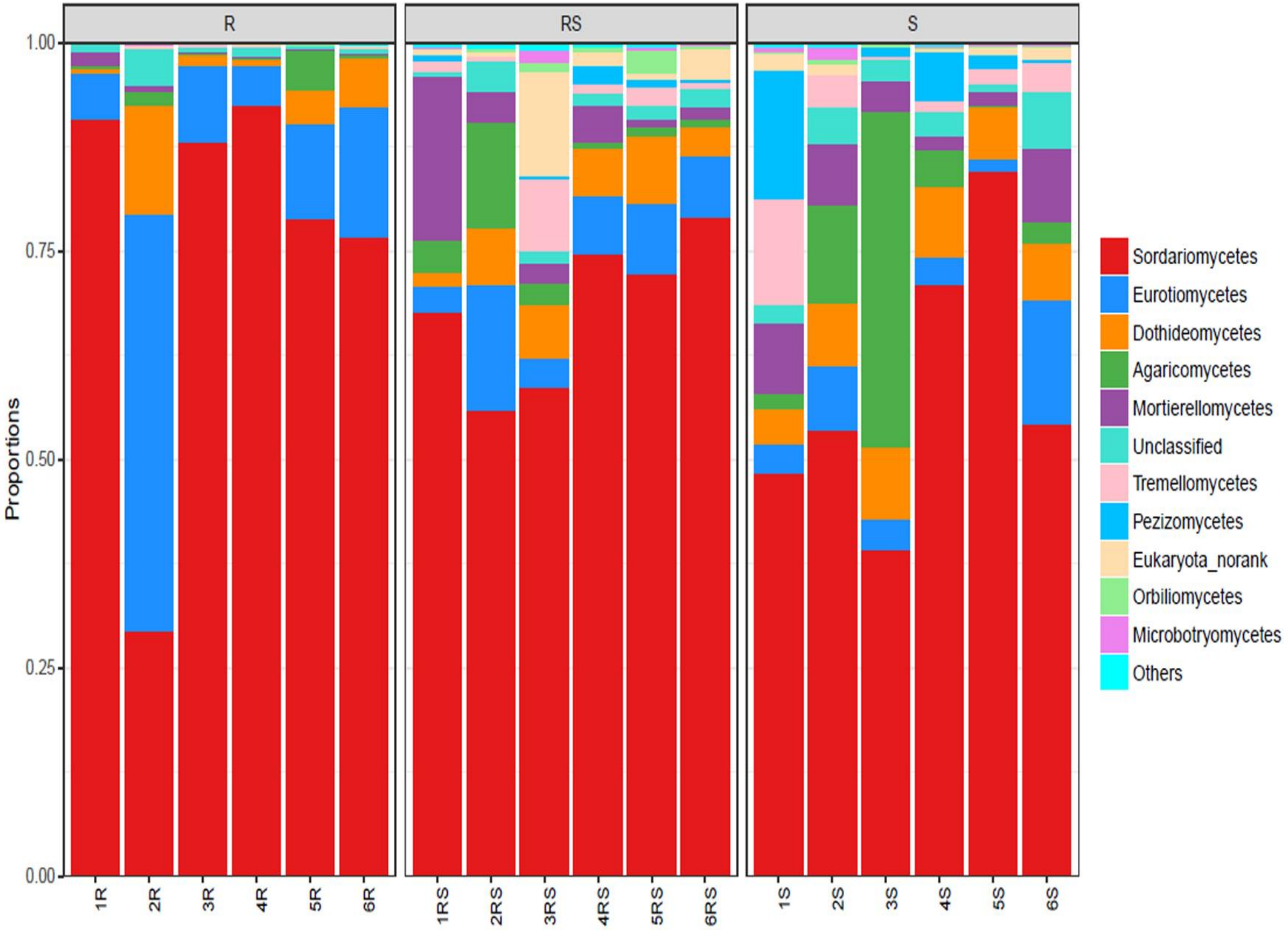

Figure S7: Phylum of soil and root fungal community composition of different biochar amendments in orchard (Figure 3A).

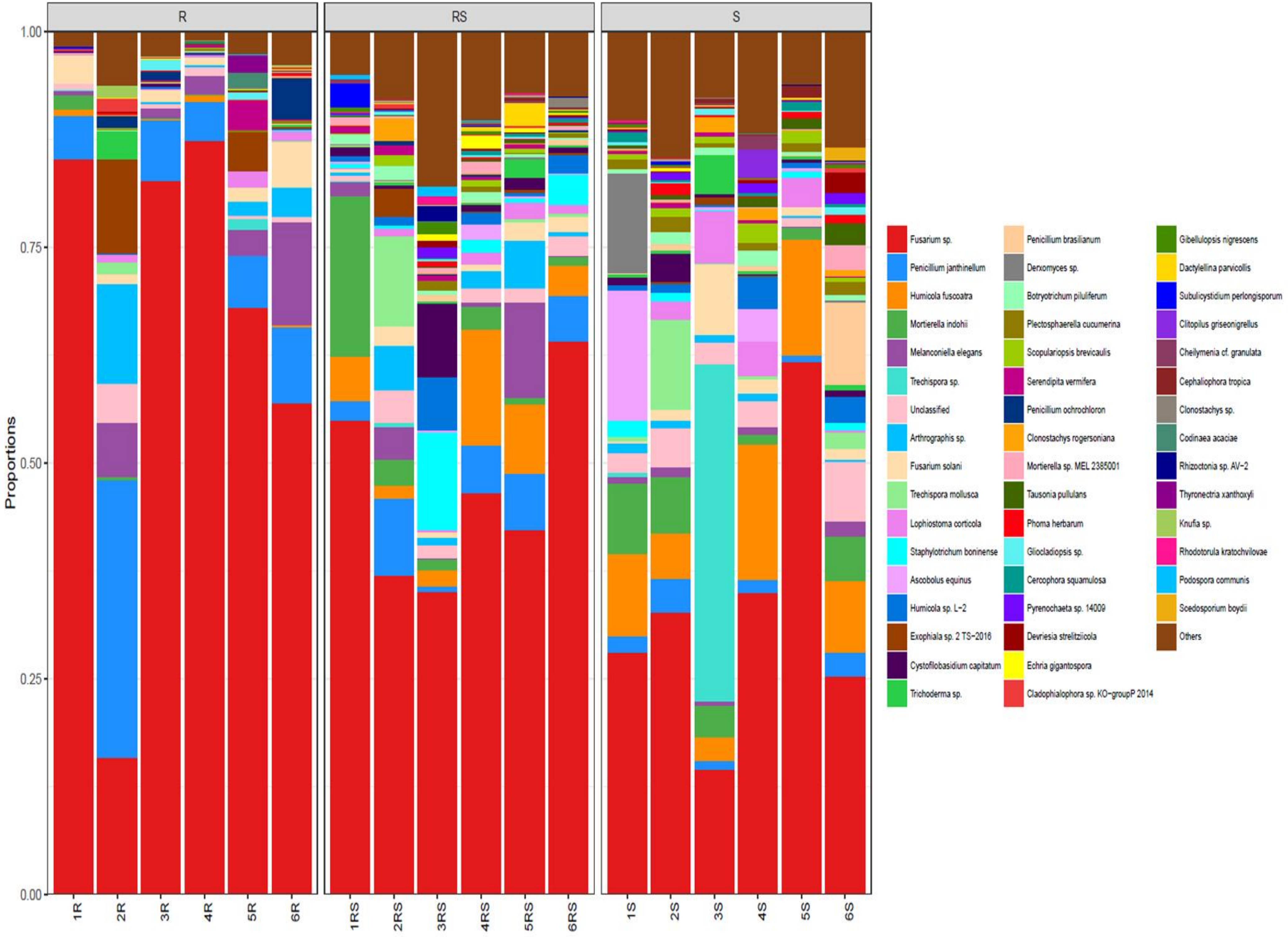

Figure S8: Genera of soil and root fungal community composition of different biochar amendments in orchard (Figure 3D)

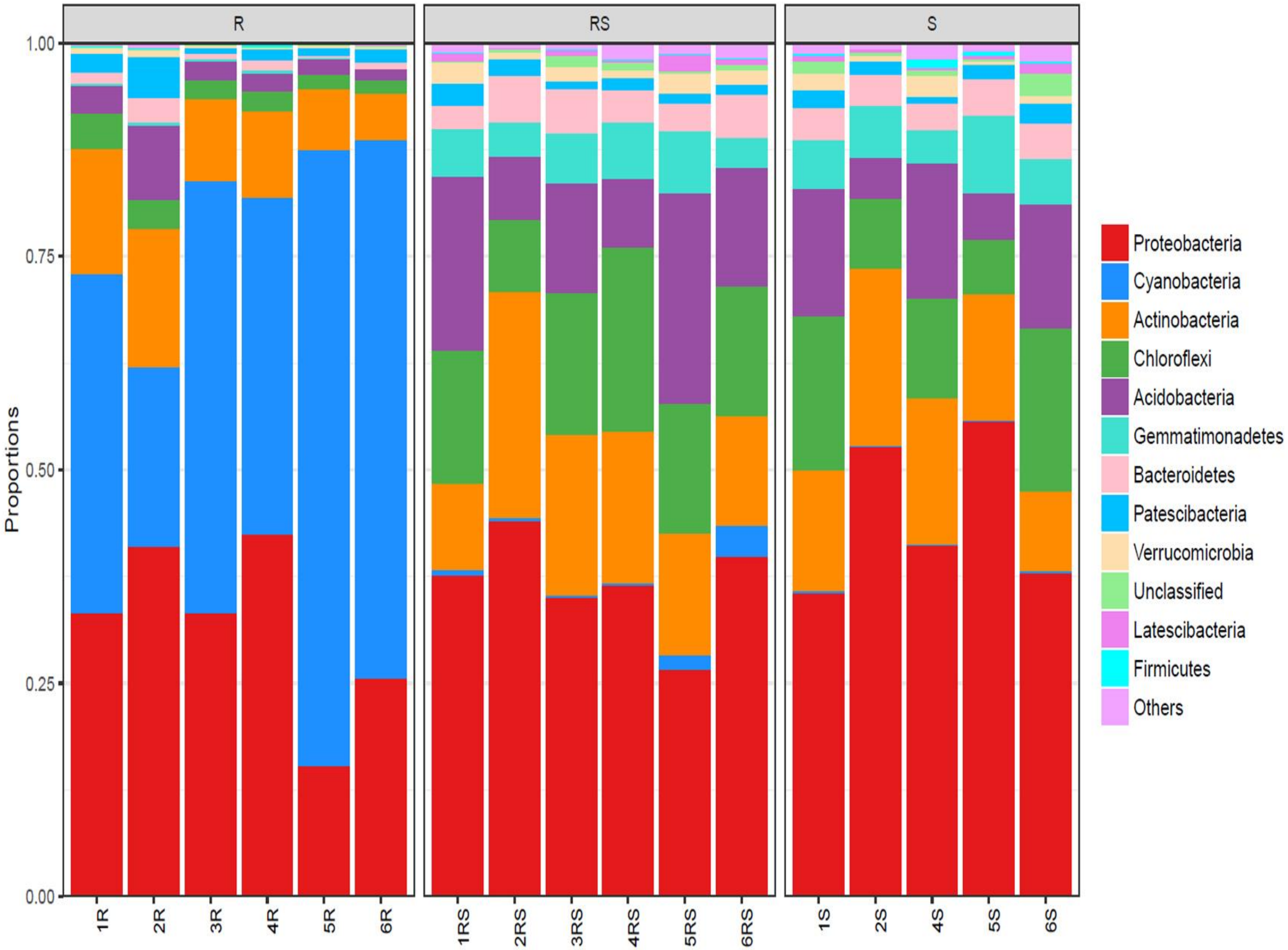

Figure S9: Phylum of soil and root bacterial community composition of different biochar amendments in orchard (Figure 3B)

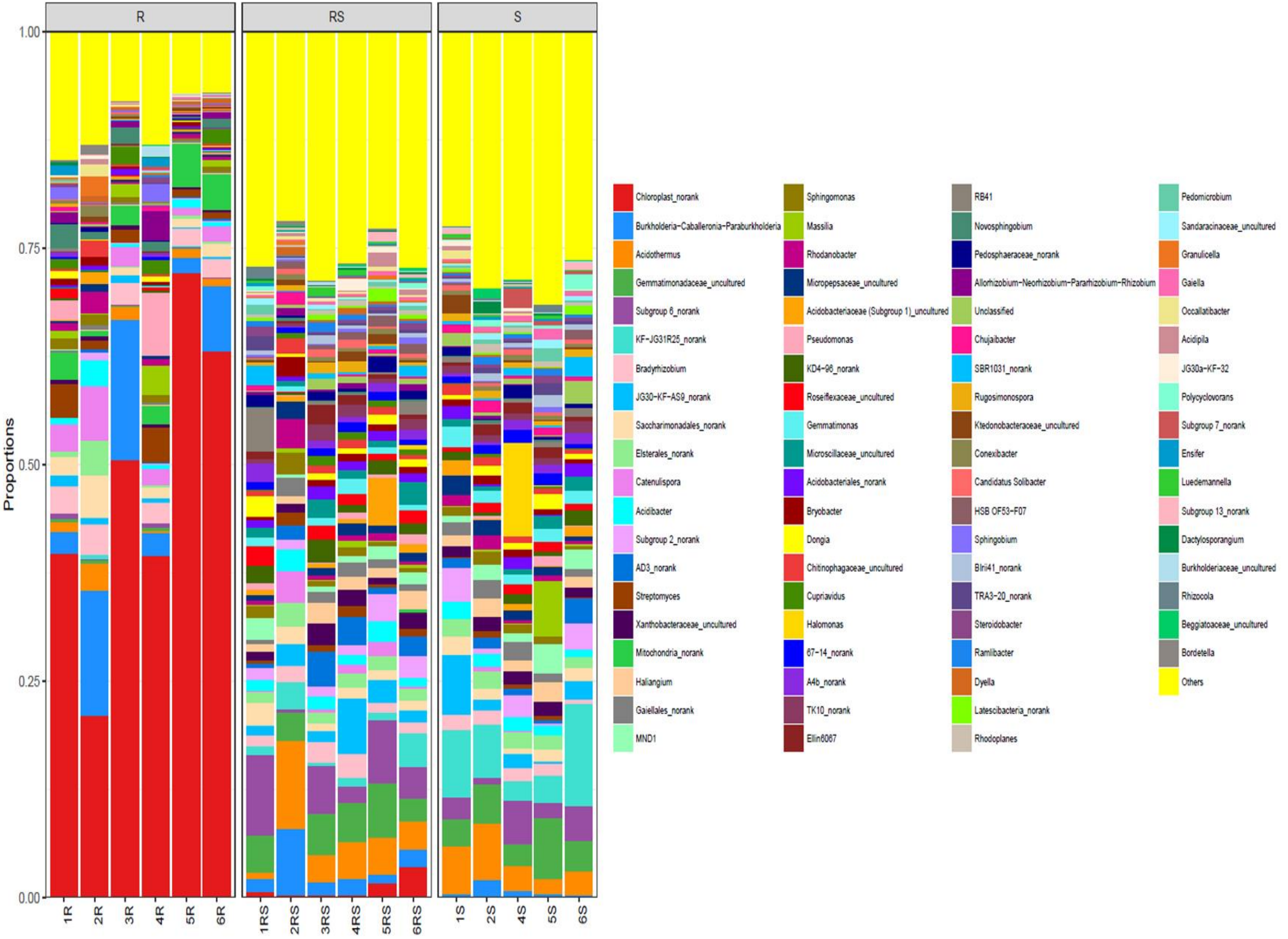

Figure S10: Genera of soil and root bacterial community composition of different biochar amendments in orchard (Figure 3E)

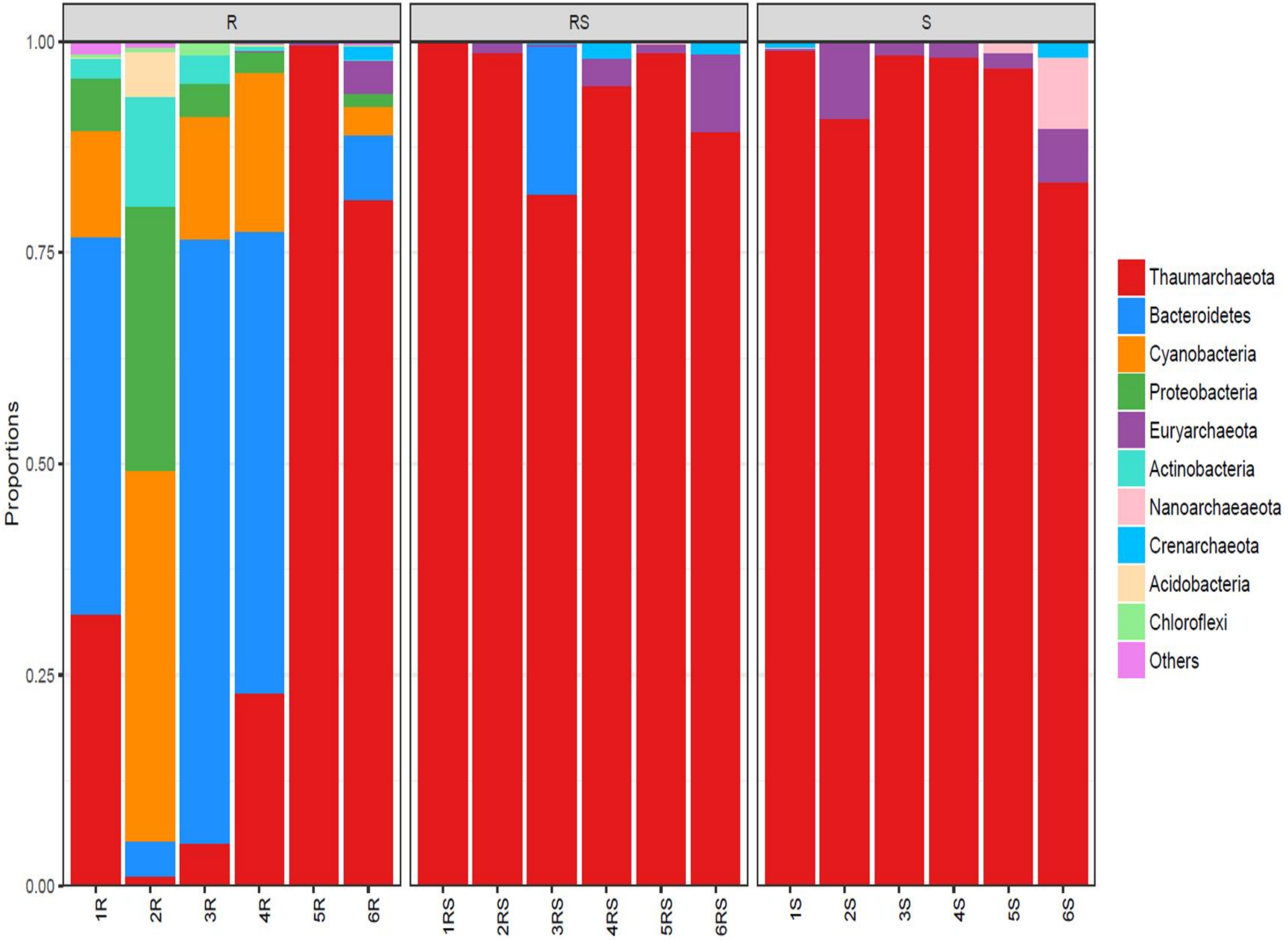

Figure S11: Phylum of soil and root Archaea community composition of different biochar amendments in orchard (Figure 3C)

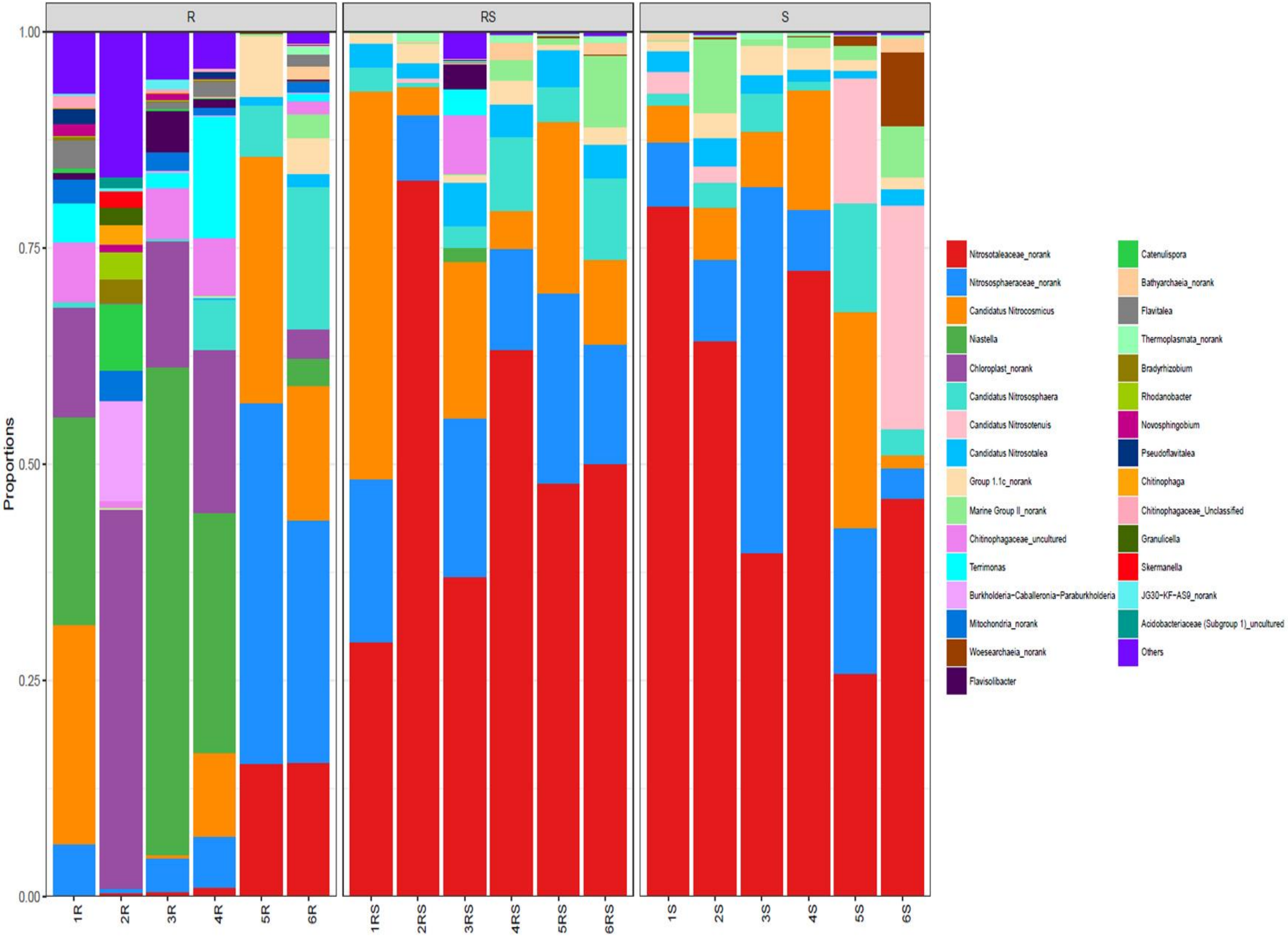

Figure S12: Genera of soil and root Archaea community composition of different biochar amendments in orchard (Figure 3F)
